# High-Throughput Sequencing of Six Bamboo Chloroplast Genomes: Phylogenetic Implications for Temperate Woody Bamboos (Poaceae: Bambusoideae)

**DOI:** 10.1371/journal.pone.0020596

**Published:** 2011-05-31

**Authors:** Yun-Jie Zhang, Peng-Fei Ma, De-Zhu Li

**Affiliations:** 1 Key Laboratory of Biodiversity and Biogeography, Kunming Institute of Botany, Chinese Academy of Sciences, Kunming, Yunnan, People's Republic of China; 2 Plant Germplasm and Genomics Center, Germplasm Bank of Wild Species, Kunming Institute of Botany, Chinese Academy of Sciences, Kunming, Yunnan, People's Republic of China; 3 Graduate University of Chinese Academy of Sciences, Beijing, People's Republic of China; British Columbia Centre for Excellence in HIV/AIDS, Canada

## Abstract

**Background:**

Bambusoideae is the only subfamily that contains woody members in the grass family, Poaceae. In phylogenetic analyses, Bambusoideae, Pooideae and Ehrhartoideae formed the BEP clade, yet the internal relationships of this clade are controversial. The distinctive life history (infrequent flowering and predominance of asexual reproduction) of woody bamboos makes them an interesting but taxonomically difficult group. Phylogenetic analyses based on large DNA fragments could only provide a moderate resolution of woody bamboo relationships, although a robust phylogenetic tree is needed to elucidate their evolutionary history. Phylogenomics is an alternative choice for resolving difficult phylogenies.

**Methodology/Principal Findings:**

Here we present the complete nucleotide sequences of six woody bamboo chloroplast (cp) genomes using Illumina sequencing. These genomes are similar to those of other grasses and rather conservative in evolution. We constructed a phylogeny of Poaceae from 24 complete cp genomes including 21 grass species. Within the BEP clade, we found strong support for a sister relationship between Bambusoideae and Pooideae. In a substantial improvement over prior studies, all six nodes within Bambusoideae were supported with ≥0.95 posterior probability from Bayesian inference and 5/6 nodes resolved with 100% bootstrap support in maximum parsimony and maximum likelihood analyses. We found that repeats in the cp genome could provide phylogenetic information, while caution is needed when using indels in phylogenetic analyses based on few selected genes. We also identified relatively rapidly evolving cp genome regions that have the potential to be used for further phylogenetic study in Bambusoideae.

**Conclusions/Significance:**

The cp genome of Bambusoideae evolved slowly, and phylogenomics based on whole cp genome could be used to resolve major relationships within the subfamily. The difficulty in resolving the diversification among three clades of temperate woody bamboos, even with complete cp genome sequences, suggests that these lineages may have diverged very rapidly.

## Introduction

Bambusoideae is one of the largest subfamilies in the grass family (Poaceae). Within Bambusoideae, woody bamboos are distinguished from other species by their woody stems and by infrequent sexual reproduction, with flowering intervals as long as 40 to 120 years [1]. Woody bamboos are of notable economic significance and have a long history of varied uses, ranging from food to raw materials for furniture and housing around the world [2], [3]. They are primarily distributed in Asia, South America and Africa, from lowlands up to about 4000 m in altitude. Many species play important roles in their ecosystems, providing shelter or food for many specialized and rare animal species (e.g. red panda, mountain bongo) [4], [5]. The best known may be the giant panda of China, the country that is also home to the greatest diversity of woody bamboos in the world, especially in the Hengduan Mountain range of southwest China [6].

The grass family comprises about 11,000 species including the most important agricultural crops, such as rice, wheat and corn, and is one of the largest families in the angiosperms [7]. According to previous phylogenetic studies, Poaceae has been divided into several basal lineages and two major lineages including the PACMAD clade (Panicoideae, Arundinoideae, Chloridoideae, Micrairoideae, Aristidoideae, and Danthonioideae) and the BEP clade (Bambusoideae, Ehrhartoideae, and Pooideae) [8], [9], [10]. Within the BEP clade the relationships between Bambusoideae, Ehrhartoideae, and Pooideae have long been controversial [8], [10], [11]. Early work supported the sister relationship between Bambusoideae+Ehrhartoideae and Pooideae [8], while others suggested that Bambusoideae+Pooideae was sister to Ehrhartoideae [10], [11]. Bambusoideae encompasses approximately 80–90 genera and 1000–1500 species [4], [6], [12] and has been resolved as a monophyletic group, consisting of herbaceous and woody bamboos [13]. However, phylogenetic analyses have implied that woody bamboos are not monophyletic and could be geographically divided into temperate woody bamboos and tropical woody bamboos, the latter having a sister relationship to herbaceous bamboos [10], [14]. Therefore, three tribes (Arundinarieae, Bambuseae and Olyreae) were formulated in Bambusoideae [14]. Temperate woody bamboos (i.e., Arundinarieae) are highly diverse in East Asia with varied habits and complex morphological features [12], [15]. They are notorious for being taxonomically difficult and having a complicated taxonomy. Thus, a robust phylogenetic tree is still needed for temperate woody bamboo classification and elucidation of the evolutionary history of this distinct lineage. Recent phylogenetic analyses based on multiple chloroplast (cp) DNA regions have provided a moderate resolution of the relationships within Arundinarieae, which was firstly resolved to include six lineages [16] and then extended to ten major lineages [17], i.e., Bergbamboes, the African alpine bamboos, *Chimonocalamus*, the *Shibataea* clade, the *Phyllostachys* clade, the *Arundinaria* clade, *Thamnocalamus*, *Indocalamus wilsonii*, *Gaoligonshania* and *Indocalamus sinicus*. Although large DNA sequence data (totaling 9,463 bp [17] and 12,943 bp [16]) were used in the two studies, relationships among major lineages and internal relationships within lineages remained unresolved.

Chloroplasts were derived from endosymbiosis between independent living cyanobacteria and a non-photosynthetic host [18]. Each has its own genome that is usually nonrecombinant and uniparentally inherited [19]. Most higher-plant cp genomes have conserved quadripartite structure, composed of two copies of a large inverted repeat (IR) and two sections of unique DNA, which are referred to as the “large single copy regions” and “small single copy regions” (LSC and SSC, respectively) [20]. Comparative analysis of the cp genome architecture indicates that the gene order and gene content are highly conserved in most cp genomes [20]. Cp-derived DNA sequences have been widely used for phylogenetic studies of higher plants, and sometimes it is necessary to use complete cp genome sequences for resolving complex evolutionary relationships [21], [22], [23]. However, acquiring large coverage of cp genomes has typically been limited by conventional DNA sequencing. As next-generation sequencing techniques have revolutionized DNA sequencing via high-throughput capabilities and relatively low costs [24], it is now more convenient to obtain cp genome sequences and extend gene-based phylogenetics to phylogenomics.

While 18 complete cp genomes belonging to five Poaceae subfamilies (Anomochlooideae, Bambusoideae, Ehrhartoideae, Panicoideae and Pooideae) have been available in NCBI GenBank (http://www.ncbi.nlm.nih.gov/genbank), no temperate woody bamboo cp genome has been sequenced. To further elucidate the phylogenetic relationships in the BEP clade and especially to examine whether the complex evolutionary relationships of temperate woody bamboos could be resolved by cp phylogenomics, we completed six woody bamboo cp genomes using next-generation Illumina sequencing-by-synthesis technology [24]. The six sequenced bamboos included one species of tropical woody bamboos, or Bambuseae, and five species of temperate woody bamboos, or Arundinarieae, which belong to three major lineages according to a recent phylogenetic study [17]. We then investigated the evolutionary patterns of grass cp genomes with an emphasis on Bambusoideae, and performed phylogenomic analyses based on a data set composed of 24 complete cp genomes in Poaceae. To date, this is the largest whole cp genome data set used in grass phylogenetic inference and an initial attempt to resolve the complex evolutionary relationships in Arundinarieae using whole cp genomes.

## Results and Discussion

### Genome sequencing, assembly and four junctions validation

One tropical woody bamboo *Bambusa emeiensis* L. C. Chia & H. L. Fung and five temperate woody bamboos *Acidosasa purpurea* (Hsueh & T. P. Yi) P. C. Keng, *Ferrocalamus rimosivaginus* T. H. Wen, *Indocalamus longiauritus* Handel-Mazzetti, *Phyllostachys edulis* (Carrière) J. Houzeau, and *Phyllostachys nigra* var. *henonis* (Mitford) Stapf ex Rendle were chosen for sequencing mainly due to their economic significance (i.e., *B. emeiensis* and moso bamboo (*P. edulis*) [6]) and phylogenetic positions in recent studies [16], [17]. According to Zeng et al. [17], *I. longiauritus*, *P. edulis* and *P. nigra* var. *henonis* were in the *Phyllostachys* clade, and *A. purpurea* and *F. rimosivaginus* belonged to the *Arundinaria* clade and the *Shibataea* clade, respectively. Illumina paired-end (73 bp and 75 bp) sequencing produced 225 Mb of data for each species. We aligned 1,594,119 paired-end reads of each species to the published cp genome of *Dendrocalamus latiflorus* (FJ970916) [25], which was chosen as a reference genome in our study with Bowtie software [26]. 198,446 (*I. longiauritus*) to 415,874 (*F. rimosivaginus*) paired-end reads were mapped to the reference genome (Table 1). After *de novo* and reference-guided assembly as in [27] with minor changes, we obtained four complete cp genomes. The other two cp genomes had only small gaps (two gaps per genome, averaging 372 bp according to the reference genome), which were then finished by PCR sequencing. The summary of the assembled contigs showing significant identity to the reference cp genome is listed in Table 1. The N50 of contigs ranged from 607 bp to 1,560 bp and the summed length of contigs for all genomes ranged from 101,015 bp to 118,081 bp. The mean coverage of the genome was from 222.1× to 441.3×. Successful recovery of these genomes supposes that ∼200× sequence coverage sufficient for assembly under this sequencing strategy and additional sequence coverage does not improve it.

**Table 1:** Summary of the chloroplast genome sequencing, assembly and features.

	*Bambusa emeiensis*	*Aciodosasa purpurea*	*Ferrocalamus rimosivaginus*	*Indocalamus longiauritus*	*Phyllostachys edulis*	*Phyllostachys nigra* var. *henonis*
Total paired-end reads	1,594,119	1,594,119	1,594,119	1,594,119	1,594,119	1,594,119
Aligned paired-end reads	211,596	370,307	415,874	198,446	209,601	263,808
Mean coverage	224.5	392.3	441.3	210.3	222.1	279.2
Number of contigs	140	219	249	167	161	184
Mean length (bp)	842	515	406	707	722	623
N50 (bp)	1560	967	607	1333	1557	1495
Sum contigs length (bp)	117,936	112,775	101,015	118,081	116,162	114,568
Number of gaps	2	0	0	0	2	0
Size (bp)	139,493	139,697	139,467	139,668	139,679	139,839
LSC length (bp)	82,988	83,273	83,091	83,273	83,213	83,234
SSC length (bp)	12,901	12,834	12,718	12,811	12,870	12,879
IR length (bp)	21,802	21,795	21,829	21,792	21,798	21,863
Number of genes	131	131	131	131	131	131
Protein-coding genes	84	84	84	84	84	84
Structure RNAs	47	47	47	47	47	47
GC content (%)	38.9	38.9	38.8	38.9	38.9	38.9
Coding regions (%)	50.7	50.6	50.4	50.6	50.6	50.6

Four junction regions between IRs and SSC/LSC in each cp genome were confirmed by PCR amplifications and Sanger sequencing using primers (Table S1) designed on the basis of the reference genome. The amplified sequences from six species totaled 20,447 bp. We compared these sequences directly to the assembled genomes, observing no nucleotide mismatches or indels. This result also validated the accuracy of our genome sequencing and assembly.

### Genome features and sequence divergence

**Figure 1:**
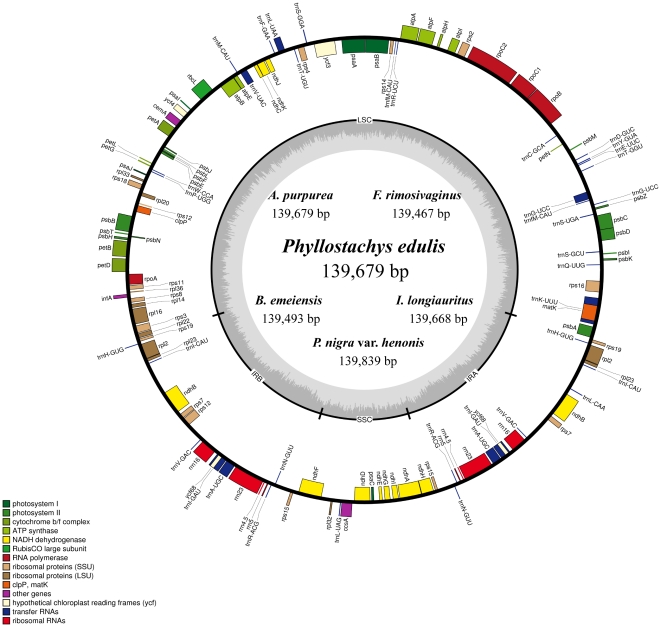
Gene map of the six woody bamboo chloroplast genomes. Genes shown outside the outer circle are transcribed clockwise and those inside are transcribed counterclockwise. Genes belonging to different functional groups are color coded. Dashed area in the inner circle indicates the GC content of the chloroplast genome.

The determined nucleotide sequences of six cp genomes ranged from 139,493 bp in *B. emeiensis* to 139,839 bp in *P. nigra* var. *henonis* (Table 1). All six cp genomes showed a typical quadripartite structure, consisting of a pair of IRs (21,792–21,863 bp) separated by the LSC (82,988–83,273 bp) and SSC (12,718–12,901 bp) regions (Table 1). They encode an identical set of 131 genes with the same gene order and gene clusters, of which 112 are unique and 19 are duplicated in the IR regions (Figure 1). The 112 unique genes include 4 ribosomal RNAs, 31 transfer RNAs and 77 protein-coding genes. Fifteen distinct genes (*rps16*, *atpF*, *rpl16*, *rpl2*, *ndhB*, *rps12*, *ndhA*, *petB*, *petD*, *trnK*(UUU), *trnG*(UCC), *trnL*(UAA), *trnV*(UAC), *trnI*(GAU), *trnA*(UGC)) contain one intron and only one gene (*ycf3*) contains two. The cp genomes consist of 50.4% to 50.7% coding regions, and the overall GC content is 38.9% for all species except for *A. purpurea* (38.8%). Altogether, these six cp genomes are highly conserved in each aspect of genome features, such as gene content and gene order, intron and GC content.

The cp genomes of these six bamboo species are also very similar in structure to those of other grasses. The grass cp genomes have been under an elevated evolutionary rate in the common grass ancestor [28] and are characterized by several structural rearrangements like inversions [29] and gene loss [30], [31], [32], [33]. Panicoideae, Pooideae and Ehrhartoideae have cp genomes with average sizes of 140,766 bp, 135,686 bp and 134,509 bp, respectively, and the cp genome of Bambusoideae averages the second in size: 139,561 bp, with a narrow variation of 139,350 bp to 139,839 bp. The grass cp genomes are smaller than those of other monocots (which average 155,410 bp based on seven representative species). The decrease in grass cp genome size was partially due to the loss of genes such as *accD*, *ycf1*, and *ycf2*, and the elimination of introns from *clpP* and *rpoC1* [30], [31], [32], [33]. Like other grasses, we found that these six bamboo cp genomes also lack these genes and introns. Aside from these sequence contractions, a unique insertion of ∼400 bp in *rpoC2* was observed in Poaceae [34]. However, the insertion in *rpoC2* of *F. rimosivaginus* is only 93 bp compared with tobacco [35], making it the shortest documented *rpoC2* gene (4,230 bp) of Poaceae (Table S2). Therefore, we speculate that deletions may have occurred following an initial insertion during the evolution of *rpoC2* in *F. rimosivaginus*.

The gene order of grass cp genomes is distinct from that of standard angiosperm cp genomes due to three typical inversions [29], which also exist in these six bamboo cp genomes. The junction positions between IRs and single copy regions often change among various plants due to IR contraction and expansion [36], and in Poaceae the termini of two genes, *ndhH* and *ndhF*, have migrated repeatedly into and out of the adjacent IRs [37]. Nevertheless, the junctions are nearly identical in all six cp genomes with *ndhH* extending 172–195 nucleotides (data not shown) into the IR, and *ndhF* confined to the SSC region, just like most species in the BEP clade [37].

**Figure 2:**
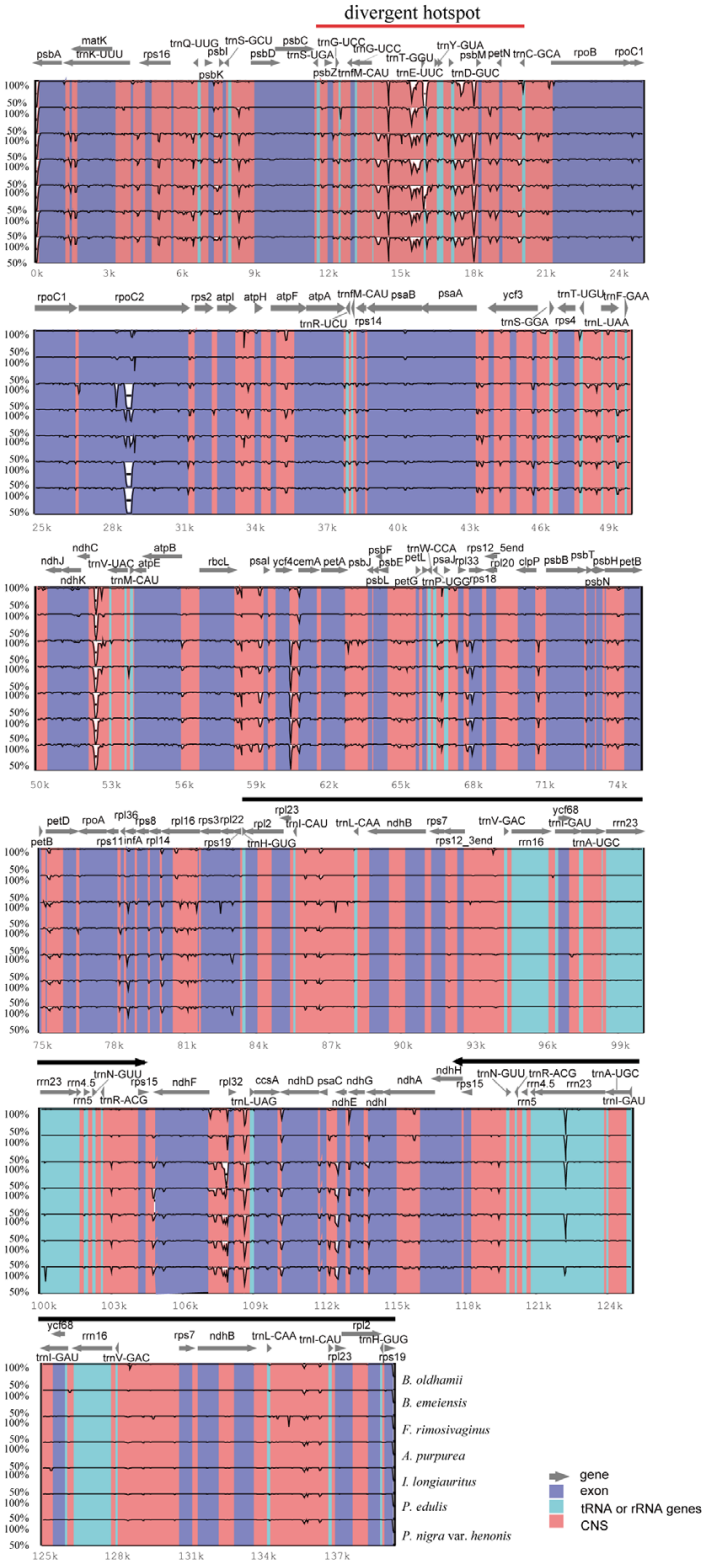
Visualization of alignment of the eight woody bamboo chloroplast genome sequences. VISTA-based identity plots showing sequence identity between six sequenced chloroplast genomes and the two published chloroplast genomes of Bambuseae, with *Dendrocalamus latiflorus* as a reference. Thick black lines show the inverted repeats (IRs) regions and red line indicates the divergent hotspot region in the chloroplast genome. Genome regions are color coded as protein coding, rRNA coding, tRNA coding or conserved noncoding sequences.

The genetic divergence is very low among cp genomes of Bambusoideae. After alignment of our six cp genomes and two other published cp genomes in Bambuseae, *Bambusa oldhamii* (FJ970915) and *D. latiflorus* [25], we plotted sequence identity using VISTA [38] with *D. latiflorus* as a reference (Figure 2). The whole aligned sequences show high similarities with only a few regions' sequence identities falling below 90%, suggesting that bamboo cp genomes are rather conservative. As expected, the IRs and coding regions are more conserved than single copy and noncoding regions, respectively. The *rpoC2* gene is an exception, with lower sequence identity due to various indels, as also found in other grasses [31], [33]. One divergent hotspot region associated with a tRNA cluster in LSC (*trnS*(UGA)-*trnC*(GCA)) region was identified (Figure 2), and this divergent hotspot has also been described in other grass cp genomes [30], [33]. The average genetic divergence of the eight bamboo species, estimated by p-distance, was only 0.009. The p-distance between Arundinarieae and Bambuseae, however, was 0.014, a much larger divergence than that within tribes (0.002 for Arundinarieae and 0.003 for Bambuseae). These values indicate that the majority of the extremely low sequence divergence in Bambusoideae mainly prevails between tribes, and that sequence divergence in Bambuseae is slightly larger than that in Arundinarieae.

### Repetitive sequences

**Figure 3:**
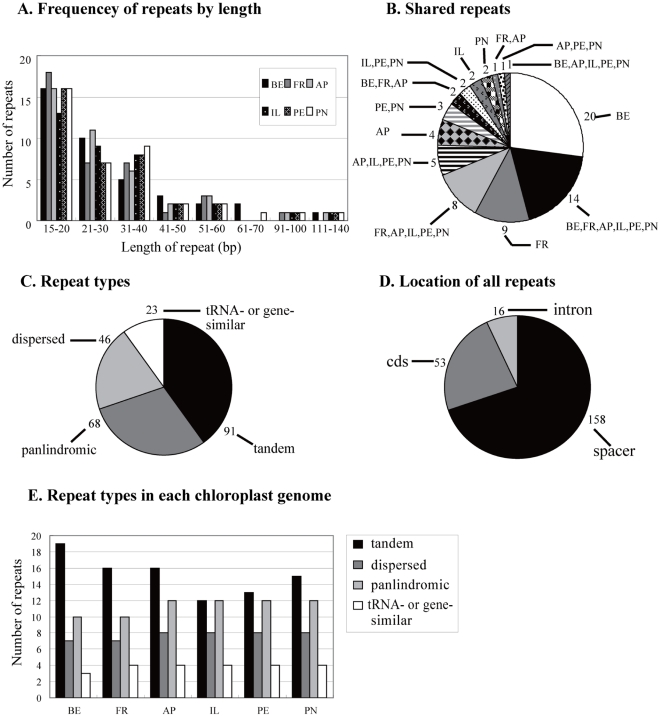
Repeat analyses. BE, *B. emeiensis*; FR, *F. rimosivaginus*; AP, *A. purpurea*; IL, *I. longiauritus*; PE, *P. edulis*; PN, *P. nigra* var. *henonis*. (A) Histogram showing the number of repeats in the six woody bamboo chloroplast genomes. (B) Summary of shared repeats among six bamboos. tRNA- or gene-similar repeats are excluded. (C) Composition of the 228 repeats from six bamboos. (D) Location of the 228 repeats from six bamboos. Repeats that occurred in two regions were counted in both. (E) Histogram showing the number of four repeat types in each bamboo chloroplast genome.

We divided repeats into three categories: dispersed, tandem and palindromic repeats. For all repeat types, the minimal cut-off identity between two copies was set to 90%. The minimal copy size investigated were 30 bp for dispersed, 15 bp for tandem and 20 bp for palindromic repeats, respectively. In all, 228 repeats were detected in six bamboo cp genomes (Table S3) using REPuter [39]. Manual verification of these identified repeats revealed that some repeats were associated with two tRNA (e.g. *trnfM*(CAU)) copies, or gene duplication (e.g. *psaA/psaB*), and these repeats may simply be due to similarity of gene functions and thus we classified them into another type—tRNA or gene similarity repeats (same procedure as was used in [40]). Tandem repeats, accounting for 39.9% of total repeats, are the most common of the four types (Figure 3C). The majority of repeats are located in noncoding regions (Figure 3D), while some are found in genes such as *infA*, *rpoC2*, *rps18* and *rps3*. 82.7% of repeats range in size between 15 bp and 40 bp (Figure 3A), although the defined smallest size is 20 bp and 30 bp for palindromic and dispersed repeats, respectively. The longest repeat is a dispersed repeat of 132 bp in *B. emeiensis*. Except for a 65 bp tandem repeat in *P. nigra* var. *henonis*, all other tandem repeats are 45 bp or shorter, while palindromic repeats occur in a narrower size range from 20 to 25 bp. Numbers of the four repeat types are similar among these six cp genomes (Figure 3E) and their overall distribution in the cp genome is highly conserved. Thus we investigated the repeats shared between species using strict criteria. Repeats that had identical lengths, and which were located in homologous regions were defined as shared repeats. Under these criteria there were 14 repeats shared by all six bamboo species and 8 repeats shared by the five woody temperate bamboos (excluding shared repeats with *B. emeiensis*) (Figure 3B). *B. emeiensis* had the most unique repeats (20), while *P. edulis* showed no unique repeats.

Repeat sequence may play a role in the rearrangement of cp genomes and generating divergent regions via illegitimate recombination and slipped-strand mispairing [31], [40]. In our sequenced genomes, divergent regions are often associated with many repeats. For example, the *rpoC2* gene contains various repeats. However, the six cp genomes are perfectly syntenic and no significant structural divergence was detected, hence we could not deduce whether these repeats in bamboo cp genomes correlated with genome rearrangement. Through the alignment of eight bamboo cp genome sequences, we found only 12 small inversions (Table S4), which were flanked by palindromic repeats. Five of the 12 inversions were presumed synapomorphic inversions shared by all members from one bamboo tribe, while all the others were probably homoplasious inversions, judging by their random distribution in the eight bamboo species. Therefore, we considered whether these small inversions may provide conflicting phylogenetic information and thus should be carefully treated when aligning sequences.

**Figure 4:**
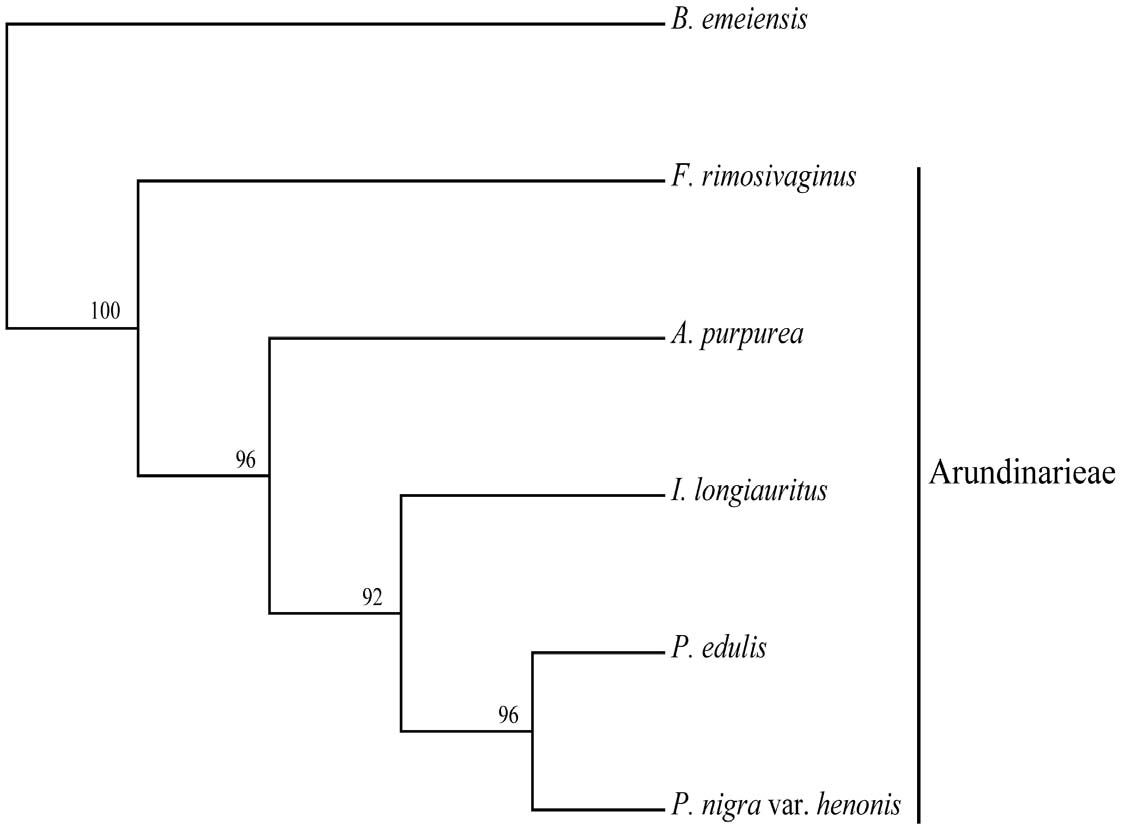
The most parsimonious tree obtained in maximum parsimony analyses of repeats in six bamboo species. Tree length is 63 steps. Consistency index is 0.968 and retention index is 0.895. Numbers at each node are bootstrap support values.

We compared the diverse repeats to determine if they could provide phylogenetic information. Maximum parsimony (MP) analyses of the identified repeats on the basis of their presence or absence in the six woody bamboo cp genomes resulted in a single most parsimonious tree with a length of 63 steps, a consistency index (CI) of 0.968, and a retention index (RI) of 0.895 (Figure 4). The resulting topology, with high bootstrap support (BS) values (≥92%) was similar to phylogenetic trees based on DNA sequences (see below). Thus, repeats in the cp genomes were found to be as useful for phylogenetic reconstruction as other genome characters such as gene content and gene order.

### Phylogenomic analyses

**Figure 5:**
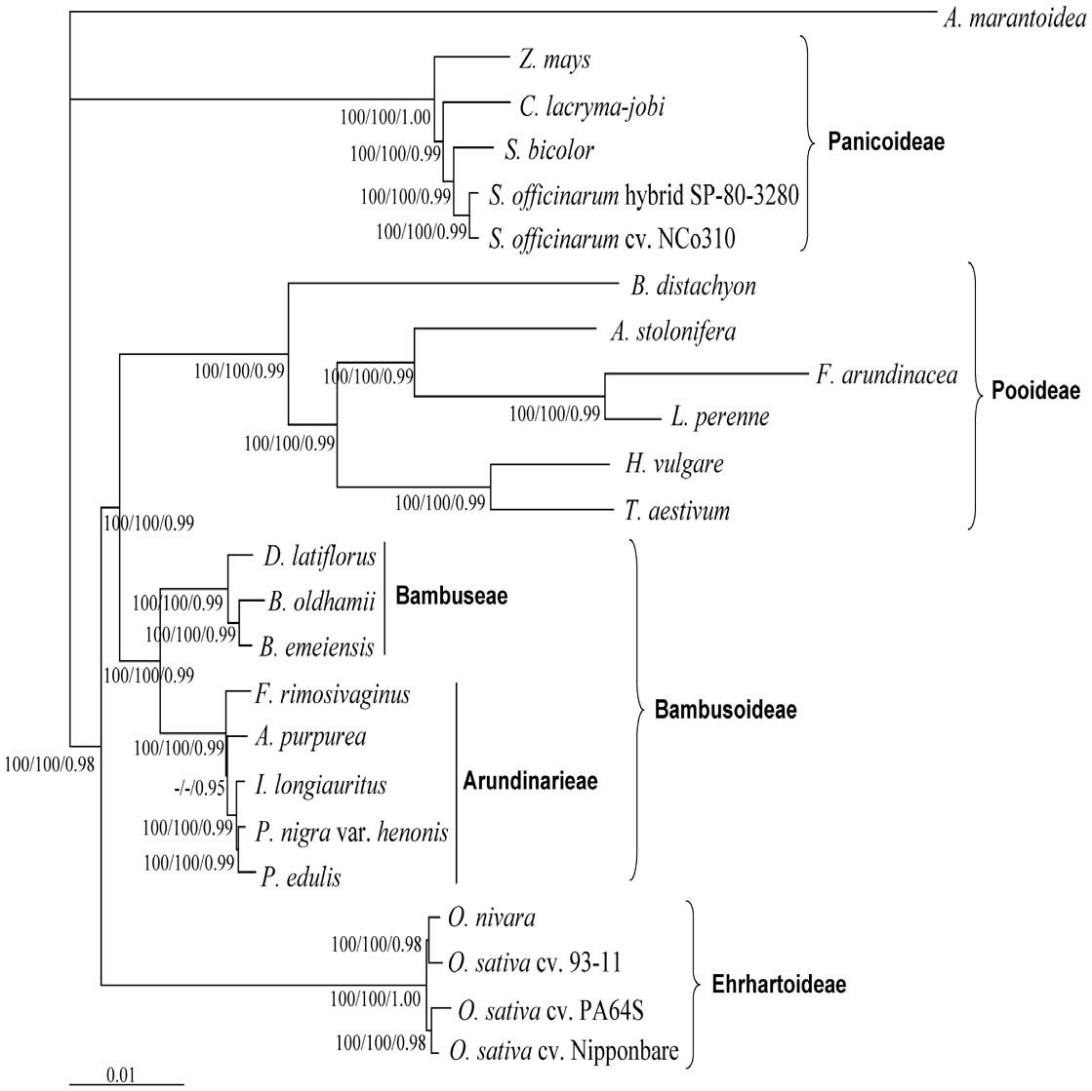
Phylogenetic relationships of 24 Poaceae accessions as determined from whole chloroplast genomes. Support values are shown for nodes as maximum parsimony bootstrap/maximum likelihood bootstrap/Bayesian inference posterior probability. Branch lengths were calculated through Bayesian analysis, and scale bar denotes substitutions per site.

**Figure 6:**
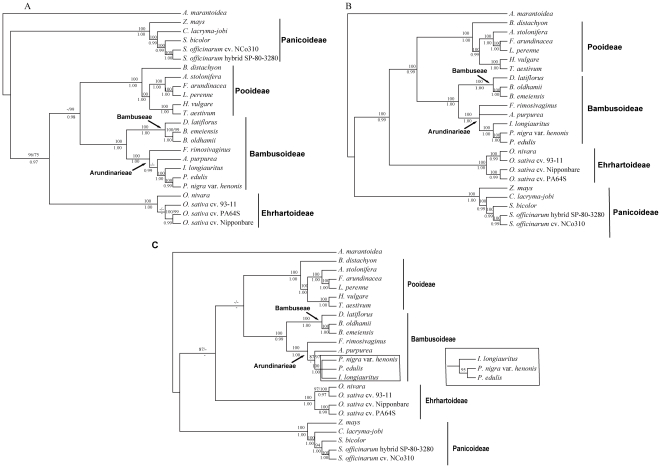
Maximum parsimony phylogram of 24 Poaceae accessions as determined from different data partitions. **A**) 74 Protein coding genes. **B**) Large single copy (LSC) region. **C**) Small single copy (SSC) region. Numbers above nodes are maximum parsimony bootstrap/maximum likelihood bootstrap support values and only one number is shown if the two values are equal. Numbers below nodes are Bayesian inference posterior probability. The inset box in the lower right indicates topological difference form maximum likelihood and Bayesian analysis.

Four data partitions (whole cp genomes, protein coding genes, the LSC region and the SSC region) (Table 2) from 24 grass cp genomes were used to construct phylogenetic trees. The SSC region had the highest percentage of parsimony informative characters (PIs) with 13.83%. However, this data partition contained the fewest PIs as the aligned sequence length was only 13,524 bp. Furthermore, SSC regions of bamboo cp genomes contained a small inversion located in the *rpl32-trnL* (UAG) intergenic spacer (Table S4) that had great influence on the branching order of *I. longiauritus*, *P. edulis* and *P. nigra* var. *henonis* (Figure S1). This inversion was considered to be of homoplasious character because of its random distribution in Bambuseae and Arundinarieae. However, the influence of the small inversion on a phylogenetic tree based on complete cp genome sequences was almost negligible. Therefore, in subsequent analysis we excluded this inversion from the data partition of SSC region. Phylogenetic trees with BS values and posterior probabilities (PP) based on the four data partitions are presented in Figures 5 and 6. The Bayesian, MP and maximum likelihood (ML) analyses yielded similar trees in each data partition and phylogenetic trees of the four data partitions were largely congruent with each other. The topological differences occurred mainly within Ehrhartoideae. Within this subfamily, three cultivated varieties of *Oryza sativa* could form a monophyletic group only when using the data partition of protein coding genes, whereas no BS or PP showed significant support for this monophyletic relationship (Figure 6A). The best resolution in phylogenetic relationships was achieved using full cp genome sequences, thus we discuss the phylogenetic relationships mainly based on Figure 5.

**Table 2:** Statistics of four data partitions used in phylogenomic analyses.

Analysis	Characteristic	Whole chloroplast genomes	Protein coding genes	LSC region	SSC region
Maximum	Aligned length (bp)	159,174	56,090	94,484	13,524
parsimony	Variable sites (%)	26,856 (16.87%)	8,241 (14.69%)	20,371 (21.56%)	3,212 (23.75%)
	Informative sites (%)	14,318 (9.00%)	4,550 (8.12%)	11,167 (11.82%)	1,868 (13.81%)
	Tree length	36,686	11,518	28,129	4,787
	Consistency index	0.838	0.803	0.835	0.799
	Retention index	0.887	0.873	0.888	0.866
	No. of nodes (BP>85%)	20	18	20	19
Maximum	−lnL	432556.519967	145981.460521	286323.112155	44316.802065
likelihood	No. of nodes (BP>85%)	20	18	20	19
Bayesian	Model	TVM+I+G	TVM+I+G	TVM+I+G	GTR+I+G
inference	No. of nodes (PP>0.95)	21	20	20	17

The BEP clade has historically been rather weakly supported since it was first identified [13], and the relationships between Bambusoideae, Pooideae and Ehrhartoideae have still not been resolved. In this study, the BEP clade was resolved as a monophyletic group with strong support (BS = 100%, PP = 0.98) and within this clade Bambusoideae was revealed to be sister to Pooideae (BS = 100%, PP = 0.99). This study was the first successful attempt to provide well-supported relationships of the three subfamilies in the BEP clade based on cp phylogenomic analyses, and the results were consistent with recent phylogenetic analyses based on selected cp DNA regions and broad sampling [10], [14]. Among these studies, Bouchenak-Khelladi *et al.* [10] performed phylogenetic analyses based on a broad representation of grass diversity, including 64 genera for the BEP clade. The overall congruence between our study and Bouchenak-Khelladi *et al.* [10] strengthens our confidence in the BEP clade relationships. The phylogenetic tree inferred from ML and Bayesian using 43 putative orthologous cDNA sequences from nuclear genome also had the same topology as this study, although the neighbor joining method yielded different topology [41]. However, further genomic and taxon sampling, especially more taxa from Ehrhartoideae, are deserved in further studies as phylogenomic analyses tends to suffer from poor sampling [42].

Woody bamboos have long been considered to be a complex and taxonomically difficult group because of their unique life history, such as predominance of asexual reproduction and infrequent flowering. Within Bambusoideae, Bambuseae and Arundinarieae were well supported (BS = 100%, PP = 0.99) as monophyletic. *B. emeiensis* was sister to *B. oldhamii* in Bambuseae and this relationship was consistent with a recent phylogenetic tree based on five cp DNA fragments [43], while it differs from phylogenetic studies which included nuclear DNA sequences in analyses [44], [45]. Triplett and Clark [46] suggested that reticulate evolution may be more significant in temperate woody bamboos than previously suspected. Natural hybridization has been reported in tropical woody bamboos as well [47]. However, whether the incongruence between phylogenies derived from cp and nuclear DNA sequences was caused by hybrid events could not be inferred from our study. Further taxon sampling and nuclear DNA sequences will likely be necessary to explain the incongruence.

Within Arundinarieae, the *Phyllostachys* clade was also supported as in the previous studies [16], [17], and further resolution was achieved among the three species in the *Phyllostachys* clade whose relationships had not previously been resolved [17]. *P. edulis* and *P. nigra* var. *henonis* were sister to *I. longiauritus* with strong support (BS = 100%, PP = 0.98). However, the sister relationship between *P. edulis* and *P. nigra* var. *henonis* was only supported by ML analyses (BS = 95%) (Figure 6C) when using the SSC region data partition. This data partition contained the shortest aligned sequence length as well as the fewest PIs, which may have led to the unsatisfactory phylogenetic resolution. From this result we concluded that extremely low genetic divergence in Arundinarieae comprised the main hindrance to the phylogenetic resolution of Arundinarieae in traditional molecular phylogenetic studies. The relationships between the *Arundinaria*, *Shibataea* and *Phyllostachys* clades were unresolved in previous studies [16], [17]. Despite our use of complete cp genome sequences, the relationships between the *Arundinaria* clade (*A. purpurea*), the *Shibataea* clade (*F. rimosivaginus*) and the *Phyllostachys* clade were only supported by Bayesian analysis, which placed the *Arundinaria* clade as sister to the *Phyllostachys* clade (PP = 0.99). Considering the phylogenetic tree (Figure 4) based on sequence repetition, the sister relationship between the *Arundinaria* and *Phyllostachys* clade seems very likely. In previous cp phylogenomic studies, it was suggested that even the entire cp genome may be insufficient to fully resolve the rapidly radiating lineages [22], [23]. Poor resolution of the diversification among the three clades may also be attributed to a rapid divergence early in the evolutionary history of Arundinarieae. However, there were only five species of Arundinarieae included in our study and insufficient taxon sampling has been known to result in unsatisfactory resolution as well. Therefore, more complete cp genome sequences of Arundinarieae are necessary to confirm the exact reason for poor resolution within the tribe. On the other hand, the increase in phylogenetic resolution indicated that phylogenomics based on complete cp genomes can be a useful alternative choice to resolve the phylogeny of complex and taxonomically difficult groups with a low rate of molecular evolution, although its ability to completely resolve the phylogeny of groups with complicated evolutionary history (i.e., involving rapid radiation and reticulate evolution) can be limited. Combining complete cp genome analysis of additional taxa with nuclear DNA sequences may eventually elucidate the evolutionary history of this distinct lineage.

Previous phylogenomic studies used common protein coding genes [21], [23]. In this study, the proportions of ingroup branches with ≥95% support were reduced by using the protein coding genes data partition and SSC region data partition, both of which both contained fewer PIs than the other two data partitions (Table 2). This result indicated that at least the LSC region was needed to provide a good resolution of these sampled taxa. Whole cp genomes which are perfectly collinear in Poaceae were proved to be more effective than common protein coding genes in our study, as evaluated by BS values and PP. Therefore, we suggest that complete cp genomes, or even just the LSC region, could be used for constructing the backbones of phylogenetic trees to resolve the relationships of main clades, as well as for solving the phylogenetic positions of some critical lineages.

**Figure 7:**
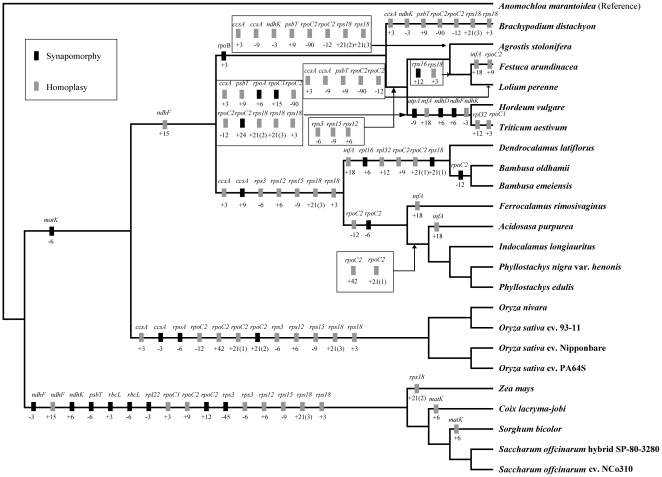
Phylogenetic distribution of exon coding indels in sampled grass accessions. Gene names are given above boxes and sizes of indels (bp) and polarity (‘+’  =  insertion, ‘−’ = deletion) are given below boxes. Polarity of mutations was determined by comparison to outgroup *Anomochloa marantoidea*. Tree topology was based on maximum parsimony analysis of complete chloroplast genomes.

Forty-five possibly informative exon indels were identified and mapped to cp genome-based phylogenetic tree (Figure 7). Of these, 25 indels mapped to monophyletic groups which have been highly supported, and thus may be synapomorphies. The remaining 20 indels may be homoplasies possibly associated with parallel mutations or back mutations during evolutionary history. There is not clear consensus about whether the indels should be used for phylogenetic analyses [48], [49], although most hesitancy against them has been based on studies using one or several DNA fragments. In our study, the 45 indels were located in 21 genes (Table S5), and they were coded and subsequently added to the protein coding gene matrix to perform MP analyses. Including indel characters in the protein coding gene matrix did not change the topology of the strict consensus tree, although it increased three nodal support values (Figure S2). Thus, we inferred that the influence of indels on our phylogenomic analyses on the basis of large data sets could be neglected. Furthermore, genes in the cp genome could contain both synapomorphic and homoplasious indels and the proportion between synapomorphic indels and homoplasious indels varied among different genes (Table S5). For example, half of the indels in the *ccsA* gene were synapomorphic, but three of four indels were homoplasious characters in *rps18* gene. Therefore, small cp genome structural changes such as indels should be carefully used in phylogenetic studies based only on several DNA fragments. Mapping the indels to species whose relationships have been well clarified could first exclude the possible homoplasious indels and thus decrease the influence of such homoplasious characters.

### Molecular marker identification

**Figure 8:**
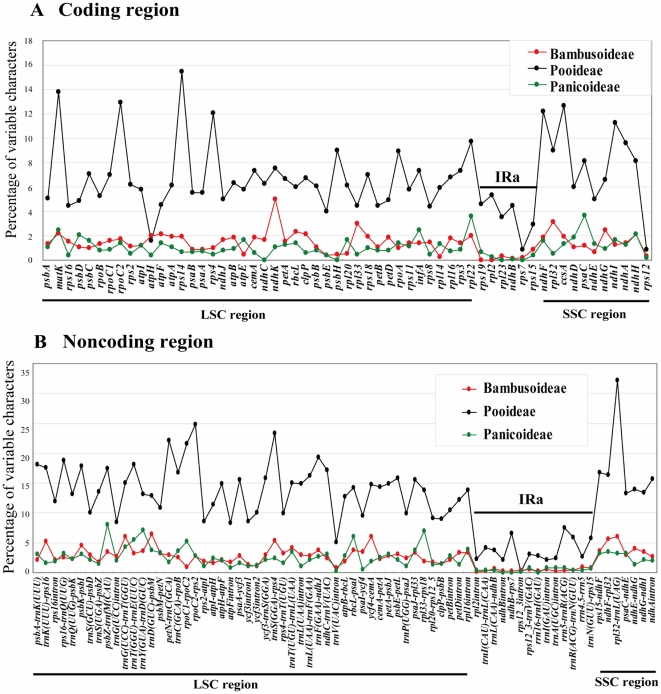
Percentage of variable characters in homologous regions among chloroplast genomes of Panicoideae, Pooideae and Bambusoideae. **A**) Coding region. **B**) Noncoding region. The homologous regions are oriented according to their locations in the chloroplast genome.

Rates of molecular evolution are linked to life history in flowering plants [50]. Woody bamboos with rather long generation times have been shown to have evolved relatively slowly in the grass family [51]. Since this low rate of molecular evolution could complicate the phylogenetic study of Bambusoideae, identifying rapidly evolving regions in bamboo cp genomes through comparative genomics is critical. We found that Pooideae accumulated more mutations in their cp genomes than Bambusoideae and Panicoideae (Figure 8) as indicated by percentage of variations (variation %). The number and distribution pattern of variable characters in coding and noncoding regions were rather different among Bambusoideae, Pooideae and Panicoideae. For example, *rpl32-trnL*(UAG) accumulated more variations than other noncoding regions in Pooideae. However, it was not the most variable region (in terms of variation percentage) in the other two subfamilies. As the evolutionary pattern of each region is different in the three subfamilies, it is more reasonable to select rapidly evolving regions for phylogenetic studies specific to each subfamily.

**Figure 9:**
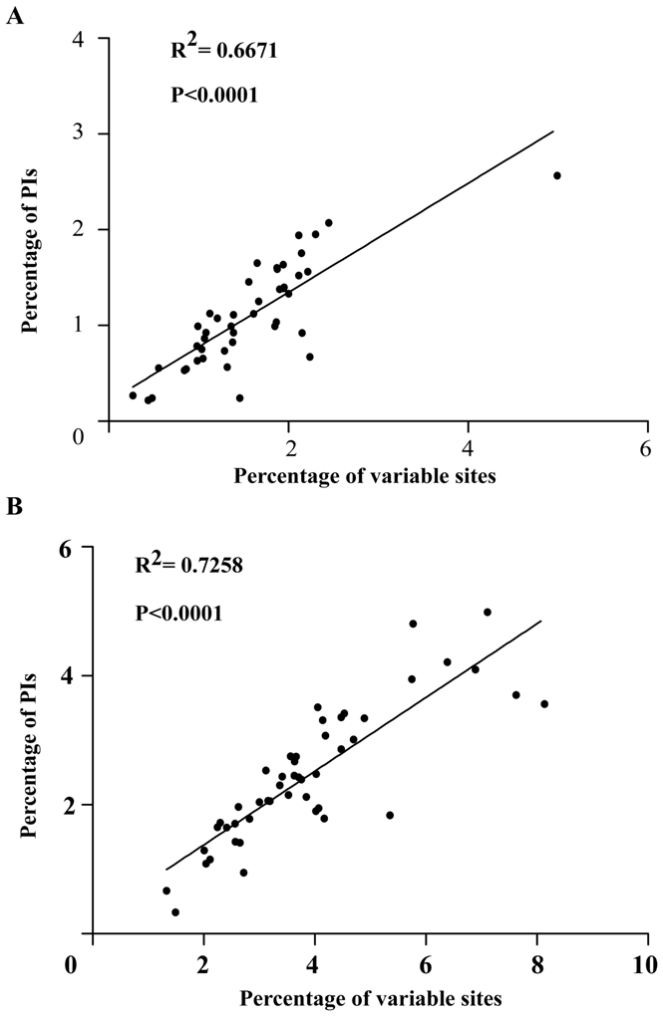
Linear relationship between percentages of parsimony informative characters (PIs) and percentages of variable sites. **A**) Coding region. **B**) Noncoding region. The fitted slope from linear regression analysis is shown.

In Bambusoideae, the proportion of variability in noncoding regions ranged from 1.33% to 8.14% and the mean value was 3.86%, which was twice as much as in the coding regions (1.57% on average). Correlation analysis revealed a significant positive linear relationship between percentages of PIs and percentages of variable sites in coding (R^2^ = 0.6671, P<0.001) and noncoding (R^2^ = 0.7258, P<0.001) regions as expected, respectively (Figure 9). Therefore, we choose the twenty most variable noncoding regions as potential molecular markers for our bamboo phylogenetic study. The variations of twenty noncoding regions exceeded 4%, and 14 of them had a percentage of PIs that exceeded 3% (Table S6). The 20 noncoding regions identified in this study are listed here from high to low relative genetic divergence: *trnD*(GUC)-*psbM*, *ycf4-cemA*, *trnG*(UCC)-*trnT*(GGU), *ndhF-rpl32*, *rpl32-trnL*(UAG), *trnK*(UUU)-*rps16*, *psbK-psbI*, *ycf3-trnS*(GGA), *trnT*(UGU)-*trnL*(UAA), *psbZ-trnfM*(CAU), *rbcL-psaI*, *psaC-ndhE*, *trnT*(GGU)-*trnE*(UUC), *trnY*(GUA)-*trnD*(GUC), *rps15-ndhF*, *trnL*(UAA)-*trnF*(GAA), *trnF*(GAA)-*ndhJ*, *rpl16* intron, *psaI-ycf4*, *psaA-ycf3*. Five of them are located in SSC region (*ndhF-rpl32*, *rpl32-trnL*(UAG), *psaC-ndhE*, *rpl16* intron) and *rps15-ndhF* is in the IRb-SSC junction. Among these regions, *rpl32-trnL*(UAG), *trnK*(UUU)-*rps16*, *trnT*(UGU)-*trnL*(UAA), *trnT*(GGU)-*trnE*(UUC), *trnY*(GUA)-*trnD*(GUC) and *psaA-ycf3* have been used in bamboo phylogenetic studies, and proved to be able to provide relatively more informative characters. However, determining whether the other 14 regions could be applied to bamboo phylogenetic analyses requires further study.

### Conclusion

In summary, here we completed six woody bamboo cp genomes including one species of Bambuseae and five species of Arundinarieae using Illumina sequencing-by-synthesis technology. These finished cp genomes may facilitate the development of biotechnological applications for these economically important woody bamboos, and provide additional information about the evolutionary history of the whole grass family. These cp genomes are highly conserved relative to other sequenced grass cp genomes. They possess several classes of repeat sequences, whose distribution and types are highly similar between sequenced cp genomes, which could provide phylogenetic information for resolving evolutionary relationships. Phylogenomic analyses based on 24 complete cp genomes from the grass family provide strong support for the placement of Bambusoideae in a sister relationship to Pooideae. Furthermore, all the relationships within Bambusoideae are well resolved with high BS and PP support, except for one node showing support only from Bayesian inference. The resolution decreased when using the relatively small SSC region data set, indicating that extremely low genetic divergence is a major hindrance for the phylogenetic resolution of Arundinarieae. Thus, we have shown cp phylogenomics to be an efficient way for resolving this difficult phylogeny. However, even using whole cp genomes the relationships between the three clades of Arundinarieae are only supported by Bayesian analysis, suggesting that temperate woody bamboos may have diverged rapidly early in their evolutionary history.

## Materials and Methods

### Ethics statement

The six sampled species were grown in Kunming Botanical Garden of the Kunming Institute of Botany among which *F. rimosivaginus* and *A. purpurea* were introduced from Jinping County of Yunnan Province, SW China in April and May 2008. The voucher specimens for the six sampled bamboos were all deposited at the Herbarium of Kunming Institute of Botany (KUN) and the collectors and numbers are Zhang 08023 for *A. purpurea*, MPF 10170 for *B. emeiensis*, Zhang 08019 for *F. rimosivaginus*, MPF 10168 for *I. longiauritus*, MPF 10163 for *P. edulis*, and MPF 10172 for *P. nigra* var. *henonis*. A field collection permit was obtained from the Forestry Department of Yunnan Province, China (permit number 2007-32).

### DNA Sequencing, genome assembly, and validation

We collected 50–100 g of fresh leaves from each species for cp DNA isolation using an improved extraction method that includes high ionic strength buffer at low pH (3.60) buffer [20], [52]. We used 5 µg of purified DNA for fragmentation by nebulization with compressed nitrogen gas, and constructed short-insert (500 bp) libraries following the manufacturer's protocol (Illumina). DNA from the different species was indexed by tags and pooled together in one lane of Illumina's Genome Analyzer for sequencing at Beijing Genomics Institute (BGI) in Shenzhen, China.

The raw sequence reads included non-cp DNA, and to determine the proportion of cpDNA we mapped sequence reads to the *D. latiflorus* cp genome using Bowtie with paired-end alignment and a maximum of 3 mismatches (-v = 3). Subsequently, three steps were used to assemble the cp genomes as in [27]. First, we assembled raw sequence reads into contigs with a minimum length of 100 bp using SOAPdenovo [53] with an overlap length of 31 bp. Second, contigs were aligned to the reference genome using BLAST (http://blast.ncbi.nlm.nih.gov/), and aligned contigs (≥90% similarity and query coverage) were ordered according to the reference genome. Third, gaps between the *de novo* contigs were replaced with consensus sequences of raw reads mapped to the reference genome.

Also based on the reference genome, we designed 5 and 4 primer pairs for closing gaps and verification of the four junctions between the single-copy segments and IRs (primers in Table S1), respectively. PCR products were sequenced using standard Sanger protocols on ABI 3730 xl instruments. Sanger sequences and assembled genomes were aligned using MEGA 4.0 [54] to determine if there were any differences.

### Genome annotation and repeat analysis

Annotation of the sequenced genomes was performed using DOGMA [55], coupled with manual selections for start and stop codons and for intron/exon boundaries. We calculated the average cp genome size of subfamilies in Poaceae on the basis of the species listed in Table 3. We estimated the monocot mean cp genome size based on *Acorus calamus* (AJ879453) [56], *Dioscorea elephantipes* (EF380353) [57], *Lemna minor* (DQ400350) [58], Oncidium Gower Ramsey (GQ324949) [59], *Phalaenopsis aphrodite* (AY916449) [60], *Phoenix dactylifera* (GU811709), and *Typha latifolia* (GU195652) [61]. The *rpoC2* sequences from the grass family were aligned to the *rpoC2* sequences of tobacco by MEGA 4.0 to determine the insertion size in the gene. We downloaded *B. oldhamii* and *D. latiflorus* cp genomes sequences from GenBank, and multiple alignments of eight bamboos cp genomes were made using MAFFT version 5 [62]. Full alignments with annotations were visualized using the VISTA viewer. The genetic divergence represented by p-distance was calculated by MEGA 4.0 with species of Arundinarieae as one group and those of Bambuseae as another.

**Table 3:** Taxa included in phylogenomic analyses of the grass family.

Classification	Taxon	GenBank	Reference
Basal lineage			
Anomochlooideae	*Anomochloa marantoidea*	GQ329703	Morris and Duvall, 2010 [69]
PACMAD clade			
Panicoideae	*Coix lacryma-jobi*	FJ261955	Leseberg and Duvall, 2009 [70]
	*Saccharum officinarum* cv. NCo310	AP006714	Asano et al., 2004 [31]
	*Saccharum officinarum* hybrid SP-80-3280	AE009947	Calsa et al., 2004 [71]
	*Sorghum bicolor*	EF115542	Saski et al., 2007 [32]
	*Zea mays*	X86563	Maier et al., 1995 [30]
BEP clade			
Pooideae	*Agrostis stolonifera*	EF115543	Saski et al., 2007 [32]
	*Brachypodium distachyon*	EU325680	Bortiri et al., 2008 [72]
	*Festuca arundinacea*	FJ466687	Cahoon et al., 2010 [73]
	*Hordeum vulgare* subsp. *Vulgare*	EF115541	Saski et al., 2007 [32]
	*Lolium perenne*	AM777385	Diekmann et al., 2008 [74]
	*Triticum aestivum*	AB042240	Ogihara et al., 2002 [75]
Ehrhartoideae	*Oryza nivara*	AP006728	Shahid et al., 2004 [76]
	*Oryza sativa* cv. 93-11	AY522329	Tang et al., 2004 [77]
	*Oryza sativa* cv. Nipponbare	X15901	Hiratsuka et al., 1989 [78]
	*Oryza sativa* cv. PA64S	AY522331	Tang et al., 2004 [77]
Bambusoideae			
Bambuseae	*Bambusa emeiensis*	HQ337797	Current study
	*Bambusa oldhamii*	FJ970915	Wu et al., 2009 [25]
	*Dendrocalamus latiflorus*	FJ970916	Wu et al., 2009 [25]
Arundinarieae	*Asidosasa purpurea*	HQ337793	Current study
	*Ferrocalamus rimosivaginus*	HQ337794	Current study
	*Indocalamus longiauritus*	HQ337795	Current study
	*Phyllostachys edulis*	HQ337796	Current study
	*Phyllostachys nigra* var. *henonis*	HQ154129	Current study

We determined the three types of repeats, dispersed, tandem and palindromic, by first applying the program REPuter and then manually filtering the redundant output of REPuter. Gap size between palindromic repeats was restricted to a maximal length of 3 kb. Overlapping repeats were merged into one repeat motif whenever possible. A given region in the genome was designated as only one repeat type, and tandem repeat was prior to dispersed repeat if one repeat motif could be identified as both tandem and dispersed repeats. For coding, each repeat present in a given genome was ‘1’ and those absent were labeled as ‘0’. We performed MP analyses of this matrix using PAUP*4.0b10 [63] to implement exhaustive tree searches. Non-parametric bootstrap analysis was conducted under 1,000 replicates with TBR branch swapping.

### Phylogenomic analyses

The six bamboo cp genome sequences, and nucleotide sequence of the 18 publicly available grass cp genomes (Table 3) were aligned using the program MAFFT version 5 and adjusted manually where necessary. The unambiguously aligned DNA sequences were used for phylogenetic tree construction. In order to examine the phylogenetic utility of different regions, phylogenetic analyses were performed based on the following data set: (1) the complete cp DNA sequences; (2) a set of 74 common protein coding genes; (3) the large single copy region; and (4) the small single copy region. MP analyses were performed with PAUP*4.0b10. Heuristic tree searches were conducted with 1,000 random-taxon-addition replicates and tree bisection-reconnection (TBR) branch swapping, with “multrees” option in effect. Non-parametric bootstrap analysis was conducted under 1,000 replicates with TBR branch swapping. Maximum likelihood (ML) analyses were implemented in RAxML version 7.2.6 [64]. RAxML searches relied on the general time reversible (GTR) model of nucleotide substitution with the gamma model of rate heterogeneity. Non-parametric bootstrapping as implemented in the “fast bootstrap” algorithm of RAxML used 1,000 replicates. Bayesian analyses were performed using the program MrBayes version 3.1.2 [65]. The best-fitting models were determined using the Akaike Information Criterion [66] as implemented in the program Modeltest 3.7 [67]. The Markov chain Monte Carlo (MCMC) algorithm was run for 200,000 generations with trees sampled every 10 generations for each data partition. The first 25% of trees from all runs were discarded as burn-in, and the remaining trees were used to construct majority-rule consensus tree. In all analyses, *Anomochloa marantoideae* was set as outgroup and all gaps introduced by the alignment were excluded.

Exon indels were mapped onto the phylogenetic tree determined by MP analyses of the whole cp genomes alignment through parsimony mapping using Mesquite version 2.7 (Maddison and Maddison, http://mesquiteproject.org).

### Molecular marker identification

To examine if the different cp genome regions evolved following a unique pattern in each subfamily, both the coding and noncoding regions longer than 200 bp were compared among taxa from Panicoideae, Pooideae and Bambusoideae. For each subfamily, homologous regions of cp genomes were aligned using MAFFT version 5 and manual adjustments were made where necessary. Subsequently, the percentage of variable characters for each region in each subfamily was calculated. Because the aim was to determine whether the evolution pattern of each region was distinct in each subfamily, only numbers of nucleotide substitutions were considered. Eight bamboo cp genomes were used to identify rapidly evolving molecular markers which may be used for bamboo phylogenetic studies. As the IR regions accumulate point mutations more slowly than do the single copy regions, only fragments from single copy regions were considered. Molecular fragments of coding regions and noncoding regions longer than 350 bp were aligned respectively. Because many indels in aligned sequences are phylogenetically informative, they were scored here as well. Then the proportion of mutational events (or variation %) for each coding and noncoding region was calculated following the modified version of the formula used in Gielly and Taberlet [68]. The proportion of mutation events = [(NS+ID)/L]×100, where NS = the number of nucleotide substitutions, ID = the number of indels, L = the aligned sequence length. As PIs are commonly used in phylogenetic analyses, the proportion of PI characters was calculated as well.

## Supporting Information

Figure S1 Phylogenetic tree derived from analysis of the SSC region (including the small inversion). Numbers at nodes indicate bootstrap support (BP) values (≥75%) from ML analyses and posterior probability (PP) support values (≥0.95) from Bayesian inference. Branch lengths were calculated through Bayesian analysis. The relationships in the box are different from those resulting from the analysis based on SSC region but excluding the small inversion.
(TIF)

Figure S2 Strict consensus tree of two parsimonious trees from the analysis of protein coding genes (gaps were coded). Tree length is 11,635 steps. Consistency index and retention index are 0.803 and 0.872, respectively. Numbers in nodes only show ≥75% bootstrap support values, and asterisks indicate increased values after adding gaps to the analysis.
(TIF)

Table S1 Primers used for gap closure and junction verification.
(DOC)

Table S2 Lengths of *rpoC2* genes and insertion sequence in *rpoC2* compared with tobacco in the grass family.
(DOC)

Table S3 Repeat sequences in the six woody bamboo chloroplast genomes.
(DOC)

Table S4 The locations and sequences of 12 small inversions in eight woody bamboo chloroplast genomes.
(DOC)

Table S5 Indels in exons of 21 genes in the grass chloroplast genomes.
(DOC)

Table S6 Summary statistics for noncoding regions in the chloroplast genomes of Bambusoideae.
(DOC)
