# Supplementary figures and images for: High-Throughput Sequencing of Six Bamboo Chloroplast Genomes: Phylogenetic Implications for Temperate Woody Bamboos (Poaceae: Bambusoideae)
Source: PLoS One. 2011 May 31;6(5):e20596. doi: 10.1371/journal.pone.0020596 (PMC3105084; doi:10.1371/journal.pone.0020596)

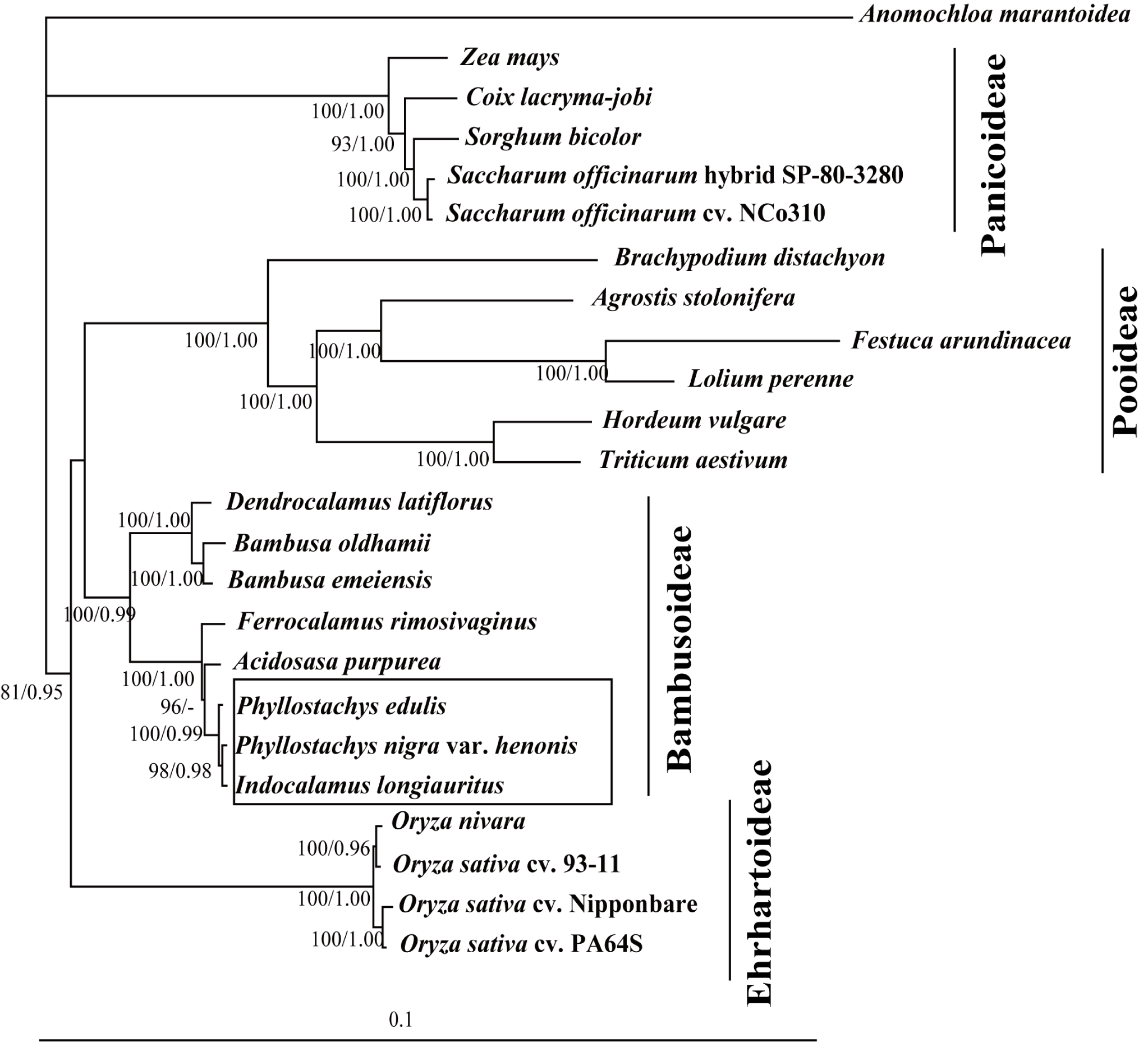

Supplement: Figure S1 — Phylogenetic tree derived from analysis of the SSC region (including the small inversion). Numbers at nodes indicate bootstrap support (BP) values (≥75%) from ML analyses and posterior probability (PP) support values (≥0.95) from Bayesian inference. Branch lengths were calculated through Bayesian analysis. The relationships in the box are different from those resulting from the analysis based on SSC region but excluding the small inversion. (TIF) [file pone.0020596.s001.tif]

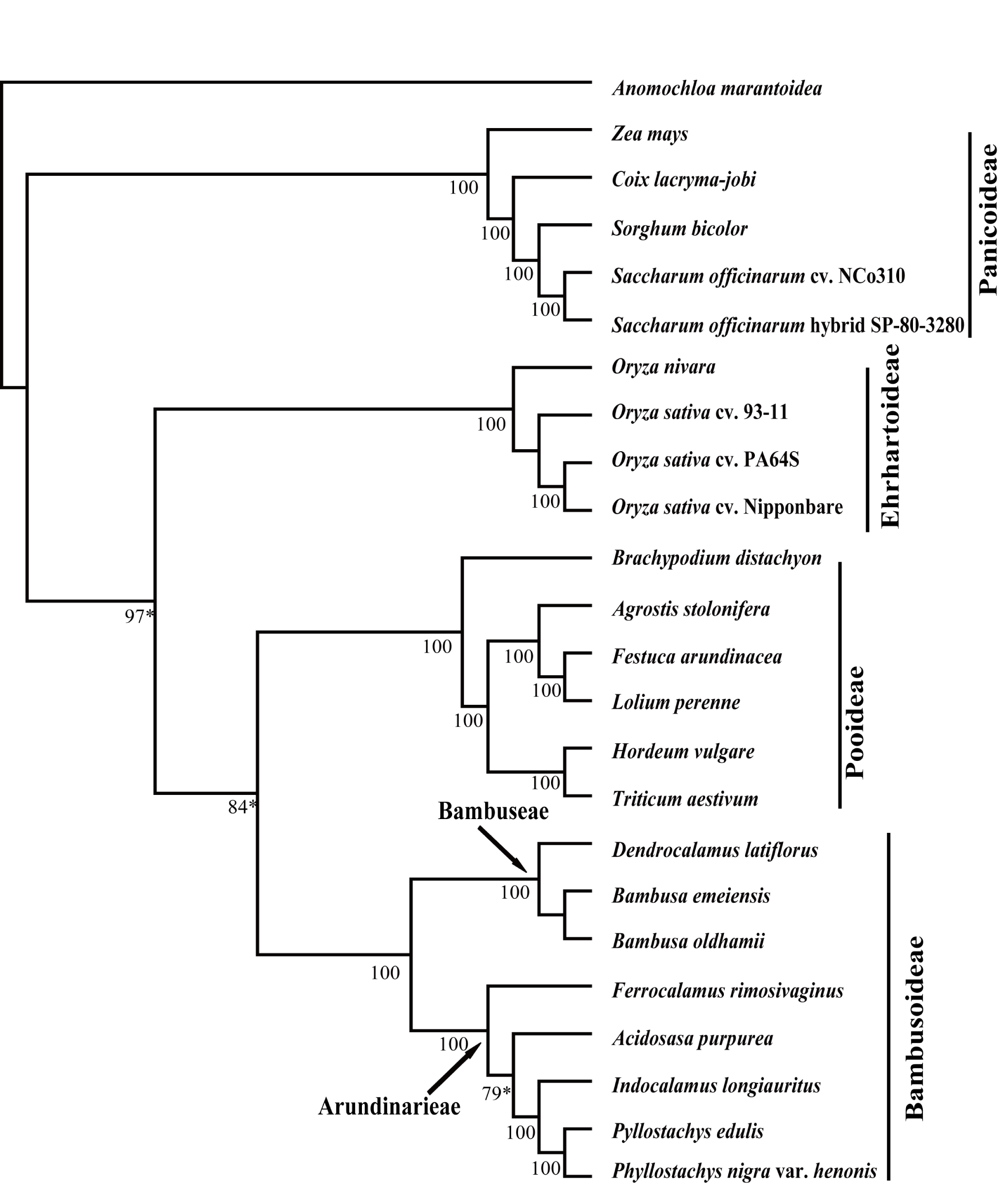

Supplement: Figure S2 — Strict consensus tree of two parsimonious trees from the analysis of protein coding genes (gaps were coded). Tree length is 11,635 steps. Consistency index and retention index are 0.803 and 0.872, respectively. Numbers in nodes only show ≥75% bootstrap support values, and asterisks indicate increased values after adding gaps to the analysis. (TIF) [file pone.0020596.s002.tif]
