# Supplementary material for: High-Throughput Sequencing of Six Bamboo Chloroplast Genomes: Phylogenetic Implications for Temperate Woody Bamboos (Poaceae: Bambusoideae)
Source: PLoS One. 2011 May 31;6(5):e20596. doi: 10.1371/journal.pone.0020596 (PMC3105084; doi:10.1371/journal.pone.0020596)
Supplement: Table S1 — Primers used for gap closure and junction verification. (DOC) [file pone.0020596.s003.doc]

**Table S1.** Primers used for gap closure and junction verification.

| **Primer^A^** | **Sequence (5’>3’)** | **Length of Gap or Amplified sequence (bp)^B^** |
| --- | --- | --- |
| 1 | R ATTCCCAGTAGGAGGGTCAA  F CTTTCGCATCGGAGGAGTAG | 76 |
| 2 | R ATTTACTGCTTCTTCTCCG  F TGGTGTTCTTCGACTGATC | 1022^C^ |
| 3 | R CTATGAGTTTGGAACCCTG  F CACCCTAAACGAAAGAAGA | 1022^C^ |
| 4 | R AATGCGATAAAATAAAGCG  F AAAGACGGGATGTCCTAAC | 49 |
| 5 | R AAAGCGAAGAACCTTACCAG  F GTGTCAAACCAAAATACCCA | 343 |
| 6 | R TACCAAGTCTGAAACCGAGTG  F AAGGTCAATATGAAGGAGGAG | 4527 |
| 7 | R TACGGAGACTTGCTTCACATT  F TACCCTGGGTATAAGAGGATT | 4575 |
| 8 | R GCATCATTCGCATACCTGT  F CGGAGACTTGCTTCACATT | 4402 |
| 9 | R AAGTGGGTAATGTTGGGGTGA  F AAATGAATCTGCTAATGAGGG | 6943 |

^A^ Primer pairs 1-3 and 4-5 were used to finish gaps in the assembly of *P. edulis* and *B. emeiensis*, respectively. Primer pairs 6, 7, 8, 9 were used to verify J_LB_, J_SB_, J_SA_ and J_LA_, respectively.

^B^ Gap lengths were determined according to the reference genome and lengths of ampified sequences were the total lengths used in the six genomes.

^C^ Closure of this gap required two primer pairs.
