# Supplementary material for: High-Throughput Sequencing of Six Bamboo Chloroplast Genomes: Phylogenetic Implications for Temperate Woody Bamboos (Poaceae: Bambusoideae)
Source: PLoS One. 2011 May 31;6(5):e20596. doi: 10.1371/journal.pone.0020596 (PMC3105084; doi:10.1371/journal.pone.0020596)
Supplement: Table S2 — Lengths of rpoC2 genes and insertion sequence in rpoC2 compared with tobacco in the grass family. (DOC) [file pone.0020596.s004.doc]

**Table S2.** Length of *rpoC2* genes and insertion sequence in *rpoC2* compared with tobacco in the grass family.

| **Taxon** | **Length of *rpoC2* (bp)** | **Length of Insertion (bp)^A^** |
| --- | --- | --- |
| **Anomochlooideae** |  |  |
| *Anomochloa marantoidea* | 4410 | 171 |
| Panicoideae |  |  |
| *Zea mays* | 4584 | 393 |
| *Coix lacryma-jobi* | 4563 | 372 |
| *Sorghum bicolor* | 4563 | 372 |
| *Saccharum officinarum* | 4605 | 414 |
| **Ehrhartoideae** |  |  |
| *Oryza nivara* | 4542 | 336 |
| *Oryza sativa* | 4542 | 336 |
| **Pooideae** |  |  |
| *Brachypodium distachyon* | 4438 | 333 |
| *Agrostis stolonifera* | 4401 | 291 |
| *Festuca arundinacea* | 4515 | 369 |
| *Lolium perenne* | 4401 | 291 |
| *Hordeum vulgare* | 4434 | 357 |
| *Triticum aestivum* | 4440 | 363 |
| **Bambusoideae** |  |  |
| *Dendrocalamus latiflorus* | 4560 | 369 |
| *Bambusa oldhamii* | 4563 | 369 |
| *Bambusa emeiensis* | 4608 | 414 |
| *A.ciodosasa purpurea* | 4575 | 390 |
| ***Ferrocalamus rimosivaginus*** | **4230** | **93** |
| *Indocalamus longiauritus* | 4620 | 435 |
| *Phyllostachys edulis* | 4554 | 369 |
| *Phyllostachys nigra* var. *henonis* *henonis henonis henonis* | 4554 | 369 |

^A^ We only inspected the insertion located nearby the middle of *rpoC2* gene.
