# Supplementary material for: High-Throughput Sequencing of Six Bamboo Chloroplast Genomes: Phylogenetic Implications for Temperate Woody Bamboos (Poaceae: Bambusoideae)
Source: PLoS One. 2011 May 31;6(5):e20596. doi: 10.1371/journal.pone.0020596 (PMC3105084; doi:10.1371/journal.pone.0020596)
Supplement: Table S3 — Repeat sequences in the six woody bamboo chloroplast genomes. (DOC) [file pone.0020596.s005.doc]

**Table S3.** Repeat sequences in the six woody bamboo chloroplast genomes.

| **Genomes^A^** | **Repeat Type** | **Length (bp)** | **Position A** | **Position B** | **Location** | **Shared Genomes** |
| --- | --- | --- | --- | --- | --- | --- |
| BE | tandem | 17 | 8315 | 8332 | *trnS*(GCU)*-psbD* |  |
| BE | tandem | 27 | 12554 | 12581 | *trnG*(UCC)*-trnfM-*(CAU) |  |
| BE | tandem | 18 | 20529 | 20547 | *trnC*(GCA)*-rpoB* | BE,FR,AP,IL,PE,PN |
| BE | tandem | 21 | 26712 | 26733 | *rpoC1-rpoC2* | BE,AP,IL,PE,PN |
| BE | tandem | 15 | 27605 | 27620 | *rpoC2* | BE,FR,AP,IL,PE,PN |
| BE | tandem | 21 | 28681 | 28702 | *rpoC2* |  |
| BE | tandem | 42 | 28789 | 28831 | *rpoC2* |  |
| BE | tandem | 45 | 29027 | 29072 | *rpoC2* |  |
| BE | tandem | 16 | 34784 | 34800 | *atpH-atpF* |  |
| BE | tandem | 31 | 52401 | 52432 | *ndhC-trnV*(UAC) |  |
| BE | tandem | 18 | 60824 | 60842 | *ycf4-cemA* | BE,FR,AP,IL,PE,PN |
| BE | tandem | 21 | 67996 | 68017 | *rps18* |  |
| BE | tandem | 17 | 70780 | 70797 | *clpP-psbB* |  |
| BE | tandem | 18 | 78617 | 78635 | *infA* | BE,FR,AP |
| BE | tandem | 45 | 82202 | 82247 | *rps3* |  |
| BE | tandem | 17 | 85687 | 85704 | *trnI*(CAU)*-trnL*(CAA) | BE,FR,AP,IL,PE,PN |
| BE | tandem | 16 | 107826 | 107842 | *ndhF-rpl32* |  |
| BE | tandem | 30 | 107941 | 107971 | *ndhF-rpl32* |  |
| BE | tandem | 16 | 112650 | 112666 | *psaC-ndhE* |  |
| BE | tRNA similarity | 20 | 12521 | 13195 | *trnG*(UCC); *trnG*(UCC) | BE,FR,AP,IL,PE,PN |
| BE | tRNA similarity | 57 | 13033 | 38269 | *trnfM*(CAU); *trnfM*(CAU) | BE,FR,AP,IL,PE,PN |
| BE | gene similarity | 38 | 40326 | 42550 | *psaB;psaA* | BE,FR,AP,IL,PE,PN |
| BE | dispersed | 51 | 12972 | 38379 | *trnG*(UCC)*-trnfM*(CAU)*; trnfM*(CAU)*-rps14* | BE,FR,AP |
| BE | dispersed | 62 | 14107 | 84767 | *trnG*(UCC)*-trnT*(GGU)*; rpl2* |  |
| BE | dispersed | 39 | 14714 | 91985 | *trnG*(UCC)*-trnT*(GGU)*; rps12_3* intron |  |
| BE | dispersed | 64 | 15887 | 15955 | *trnT*(GGU)*;trnT*(GGU) *–trnE*(UUC) |  |
| BE | dispersed | 132 | 28681 | 28861 | *rpoC2* |  |
| BE | dispersed | 39 | 28969 | 29035 | *rpoC2* | BE,FR,AP,IL,PE,PN |
| BE | dispersed | 39 | 45213 | 92659 | *ycf3*intron; *rps12_3-trnV*(GAC) | BE,FR,AP,IL,PE,PN |
| BE | panlindromic | 20 | 5 | 25 | *rps19-psbA* | BE,FR,AP,IL,PE,PN |
| BE | panlindromic | 20 | 17571 | 18301 | *trnD*(GUC)*-psbM; psbM-petN* | BE,FR,AP,IL,PE,PN |
| BE | panlindromic | 21 | 44905 | 46850 | *ycf3intron; trnS*(GGA)*-rps4* |  |
| BE | panlindromic | 20 | 58407 | 58430 | *rbcL-psaI* |  |
| BE | panlindromic | 20 | 72962 | 72987 | *psbT-psbN* | BE,FR,AP,IL,PE,PN |
| BE | panlindromic | 20 | 81059 | 81530 | *rpl16* intron |  |
| BE | panlindromic | 22 | 93378 | 93921 | *rps12_3-trnV*(GAC) | BE,FR,AP,IL,PE,PN |
| BE | panlindromic | 25 | 103983 | 104010 | *trnN*(GUU)*-rps15* | BE,FR,AP,IL,PE,PN |
| BE | panlindromic | 21 | 107486 | 107624 | *ndhF-rpl32* | BE,FR,AP,IL,PE,PN |
| BE | panlindromic | 24 | 108426 | 108456 | *rpl32-trnL*(UAG) | BE,FR,AP,IL,PE,PN |
| FR | tandem | 19 | 15474 | 15493 | *trnG*(UCC)*-trnT*(GGU) |  |
| FR | tandem | 16 | 16175 | 16191 | *trnG*(UCC)*-trnT*(GGU) | FR,AP,IL,PE,PN |
| FR | tandem | 18 | 20969 | 20987 | *trnC*(GCA)*-rpoB* | BE,FR,AP,IL,PE,PN |
| FR | tandem | 15 | 28054 | 28069 | *rpoC2* | BE,FR,AP,IL,PE,PN |
| FR | tandem | 15 | 33968 | 33983 | *atpI-atpH* | FR,AP |
| FR | tandem | 18 | 60786 | 60804 | *ycf4-cemA* | BE,FR,AP,IL,PE,PN |
| FR | tandem | 20 | 62863 | 62883 | *petA-psbJ* |  |
| FR | tandem | 19 | 67444 | 67463 | *psaJ-rpl33* |  |
| FR | tandem | 17 | 67864 | 67881 | *rpl33-rps18* | FR,AP,IL,PE,PN |
| FR | tandem | 42 | 68006 | 68048 | *rps18* | FR,AP,IL,PE,PN |
| FR | tandem | 36 | 78640 | 78676 | *infA* |  |
| FR | tandem | 18 | 81579 | 81597 | *rpl16* intron |  |
| FR | tandem | 24 | 82558 | 82582 | *rps3-rpl22* |  |
| FR | tandem | 17 | 85783 | 85800 | *trnI*(CAU)*-trnL*(CAA) | BE,FR,AP,IL,PE,PN |
| FR | tandem | 22 | 87395 | 87417 | *trnI*(CAU)*-trnL*(CAA) |  |
| FR | tandem | 20 | 107917 | 107937 | *ndhF-rpl32* |  |
| FR | tRNA similarity | 20 | 12980 | 13631 | *trnG*(UCC); *trnG*(UCC) | BE,FR,AP,IL,PE,PN |
| FR | tRNA similarity | 57 | 13464 | 38358 | *trnfM*(CAU); *trnfM*(CAU) | BE,FR,AP,IL,PE,PN |
| FR | gene similarity | 38 | 40416 | 42640 | *psaB;psaA* | BE,FR,AP,IL,PE,PN |
| FR | gene similarity | 32 | 58406 | 137103 | *rbcL-psaI*; *rpl23* | FR,AP,IL,PE,PN |
| FR | dispersed | 51 | 13403 | 38468 | *trnG*(UCC)*-trnfM*(CAU)*; trnfM*(CAU)*-rps14* | BE,FR,AP |
| FR | dispersed | 100 | 14520 | 84835 | *trnG*(UCC)*-trnT*(GGU)*;rpl2* | FR,AP,IL,PE,PN |
| FR | dispersed | 35 | 15178 | 92138 | *trnG*(UCC)*-trnT*(GGU)*; rps12_3* intron | FR,AP,IL,PE,PN |
| FR | dispersed | 54 | 16362 | 16429 | *trnT*(GGU); *trnT*(GGU)*-trnE*(UUC) | FR,AP,IL,PE,PN |
| FR | dispersed | 39 | 29091 | 29157 | *rpoC2* | BE,FR,AP,IL,PE,PN |
| FR | dispersed | 39 | 45312 | 92788 | *ycf3*intron; *rps12_3-trnV*(GAC) | BE,FR,AP,IL,PE,PN |
| FR | dispersed | 31 | 82282 | 82327 | *rps3* | BE,FR,AP,IL,PE,PN |
| FR | panlindromic | 20 | 1 | 21 | *rps19-psbA* | BE,FR,AP,IL,PE,PN |
| FR | panlindromic | 20 | 962 | 2503 | *psbA;matK* |  |
| FR | panlindromic | 20 | 18038 | 18709 | *trnD*(GUC)*-psbM; psbM-petN* | BE,FR,AP,IL,PE,PN |
| FR | panlindromic | 20 | 72978 | 73003 | *psbT-psbN* | BE,FR,AP,IL,PE,PN |
| FR | panlindromic | 22 | 93513 | 94056 | *rps12_3-trnV*(GAC) | BE,FR,AP,IL,PE,PN |
| FR | panlindromic | 25 | 104123 | 104150 | *trnN*(GUU)*-rps15* | BE,FR,AP,IL,PE,PN |
| FR | panlindromic | 22 | 107401 | 108655 | *ndhF-rpl32*; *rpl32-trnL* | FR,AP,IL,PE,PN |
| FR | panlindromic | 20 | 107444 | 107780 | *ndhF-rpl32* | FR,AP,IL,PE,PN |
| FR | panlindromic | 21 | 107606 | 107741 | *ndhF-rpl32* | BE,FR,AP,IL,PE,PN |
| FR | panlindromic | 24 | 108413 | 108443 | *rpl32-trnL*(UAG) | BE,FR,AP,IL,PE,PN |
| AP | tandem | 24 | 16072 | 16096 | *trnG*(UCC)*-trnT*(GGU) |  |
| AP | tandem | 16 | 16187 | 16203 | *trnG*(UCC)*-trnT*(GGU) | FR,AP,IL,PE,PN |
| AP | tandem | 16 | 20062 | 20078 | *petN-trnC*(GCA) | AP,IL,PE,PN |
| AP | tandem | 18 | 20951 | 20969 | *trnC*(GCA)*-rpoB* | BE,FR,AP,IL,PE,PN |
| AP | tandem | 21 | 27146 | 27167 | *rpoC1-rpoC2* | BE,AP,IL,PE,PN |
| AP | tandem | 15 | 28039 | 28054 | *rpoC2* | BE,FR,AP,IL,PE,PN |
| AP | tandem | 42 | 29241 | 29283 | *rpoC2* | AP,IL,PE,PN |
| AP | tandem | 21 | 29376 | 29397 | *rpoC2* |  |
| AP | tandem | 15 | 34313 | 34328 | *atpI-atpH* | FR,AP |
| AP | tandem | 18 | 54033 | 54051 | *trnV-trnM* |  |
| AP | tandem | 18 | 61111 | 61129 | *ycf4-cemA* | BE,FR,AP,IL,PE,PN |
| AP | tandem | 17 | 68128 | 68145 | *rpl33-rps18* | FR,AP,IL,PE,PN |
| AP | tandem | 42 | 68271 | 68313 | *rps18* | FR,AP,IL,PE,PN |
| AP | tandem | 18 | 78897 | 78915 | *infA* | BE,FR,AP |
| AP | tandem | 17 | 85965 | 85982 | *trnI*(CAU)*-trnL*(CAA) | BE,FR,AP,IL,PE,PN |
| AP | tandem | 25 | 105076 | 105101 | *rps15-ndhF* |  |
| AP | tRNA similarity | 20 | 12984 | 13629 | *trnG*(UCC)*; trnG*(UCC) | BE,FR,AP,IL,PE,PN |
| AP | tRNA similarity | 57 | 13467 | 38696 | *trnfM*(CAU)*; trnfM*(CAU) | BE,FR,AP,IL,PE,PN |
| AP | gene similarity | 38 | 40754 | 42978 | *psaB; psaA* | BE,FR,AP,IL,PE,PN |
| AP | gene similarity | 32 | 58720 | 137333 | *rbcL-psaI; rpl23* | FR,AP,IL,PE,PN |
| AP | dispersed | 51 | 13406 | 38806 | *trnG*(UCC)*-trnfM*(CAU); *trnfM*(CAU)*-rps14* | BE,FR,AP |
| AP | dispersed | 100 | 14519 | 85017 | *trnG*(UCC)*-trnT*(GGU)*;rpl2* | FR,AP,IL,PE,PN |
| AP | dispersed | 35 | 15177 | 92293 | *trnG*(UCC)*-trnT*(GGU)*; rps12_3* intron | FR,AP,IL，PE,PN |
| AP | dispersed | 54 | 16374 | 16441 | *trnT*(GGU); *trnT*(GGU)*-trnE*(UUC) | FR,AP,IL,PE,PN |
| AP | dispersed | 111 | 28112 | 29313 | *rpoC2* | AP,IL,PE,PN |
| AP | dispersed | 39 | 29421 | 29487 | *rpoC2* | BE,FR,AP,IL,PE,PN |
| AP | dispersed | 39 | 45645 | 92943 | *ycf3*intron; *rps12_3-trnV*(GAC) | BE,FR,AP,IL,PE,PN |
| AP | dispersed | 31 | 82488 | 82533 | *rps3* | BE,FR,AP,IL,PE,PN |
| AP | panlindromic | 20 | 1 | 21 | *rps19-psbA* | BE,FR,AP,IL,PE,PN |
| AP | panlindromic | 20 | 18044 | 18710 | *trnD*(GUC)*-psbM; psbM-petN* | BE,FR,AP,IL,PE,PN |
| AP | panlindromic | 23 | 18220 | 18738 | *trnD*(GUC)*-psbM; psbM-petN* | AP,IL,PE,PN |
| AP | panlindromic | 22 | 18794 | 18820 | *psbM-petN* | AP,IL,PE,PN |
| AP | panlindromic | 20 | 32012 | 34608 | *rpoC2-rps2; atpI-atpH* | AP,PE,PN |
| AP | panlindromic | 20 | 73235 | 73259 | *psbT-psbN* | BE,FR,AP,IL,PE,PN |
| AP | panlindromic | 22 | 93662 | 94205 | *rps12_3-trnV*(GAC) | BE,FR,AP,IL,PE,PN |
| AP | panlindromic | 25 | 104271 | 104298 | *trnN*(GUU)*-rps15* | BE,FR,AP,IL,PE,PN |
| AP | panlindromic | 22 | 107604 | 108940 | *ndhF-rpl32; rpl32-trnL* | FR,AP,IL,PE,PN |
| AP | panlindromic | 20 | 107647 | 107985 | *ndhF-rpl32* | FR,AP,IL,PE,PN |
| AP | panlindromic | 21 | 107809 | 107946 | *ndhF-rpl32* | BE,FR,AP,IL,PE,PN |
| AP | panlindromic | 24 | 108698 | 108728 | *rpl32-trnL*(UAG) | BE,FR,AP,IL,PE,PN |
| IL | tandem | 16 | 16139 | 16155 | *trnG*(UCC)*-trnT*(GGU) | FR,AP,IL,PE,PN |
| IL | tandem | 16 | 20006 | 20022 | *petN-trnC*(GCA) | AP,IL,PE,PN |
| IL | tandem | 18 | 20902 | 20920 | *trnC*(GCA)*-rpoB* | BE,FR,AP,IL,PE,PN |
| IL | tandem | 21 | 27098 | 27119 | *rpoC1-rpoC2* | BE,AP,IL,PE,PN |
| IL | tandem | 15 | 27991 | 28006 | *rpoC2* | BE,FR,AP,IL,PE,PN |
| IL | tandem | 42 | 29193 | 29235 | *rpoC2* | AP,IL,PE,PN |
| IL | tandem | 31 | 34122 | 34153 | *atpI-atpH* |  |
| IL | tandem | 18 | 61105 | 61123 | *ycf4-cemA* | BE,FR,AP,IL,PE,PN |
| IL | tandem | 17 | 68130 | 68147 | *rpl33-rps18* | FR,AP,IL,PE,PN |
| IL | tandem | 42 | 68273 | 68315 | *rps18* | FR,AP,IL,PE,PN |
| IL | tandem | 17 | 85967 | 85984 | *trnI*(CAU)*-trnL*(CAA) | BE,FR,AP,IL,PE,PN |
| IL | tandem | 19 | 108171 | 108190 | *ndhF-rpl32* | IL,PE,PN |
| IL | tRNA similarity | 20 | 12972 | 13613 | *trnG*(UCC); *trnG*(UCC) | BE,FR,AP,IL,PE,PN |
| IL | tRNA similarity | 57 | 13451 | 38673 | *trnfM*(CAU); *trnfM*(CAU) | BE,FR,AP,IL,PE,PN |
| IL | gene similarity | 38 | 40731 | 42955 | *psaB; psaA* | BE,FR,AP,IL,PE,PN |
| IL | gene similarity | 32 | 58727 | 137302 | *rbcL-psaI; rpl23* | FR,AP,IL,PE,PN |
| IL | dispersed | 39 | 13402 | 38795 | *trnG*(UCC)*-trnfM*(CAU); *trnfM*(CAU)*-rps14* | IL,PE,PN |
| IL | dispersed | 100 | 14503 | 85019 | *trnG*(UCC)*-trnT*(GGU)*;rpl2* | FR,AP,IL,PE,PN |
| IL | dispersed | 35 | 15156 | 92295 | *trnG*(UCC)*-trnT*(GGU)*; rps12_3 intron* | FR,AP,IL,PE,PN |
| IL | dispersed | 54 | 16326 | 16393 | *trnT*(GGU); *trnT*(GGU)*-trnE*(UUC) | FR,AP,IL,PE,PN |
| IL | dispersed | 111 | 29064 | 29265 | *rpoC2* | AP,IL,PE,PN |
| IL | dispersed | 39 | 29352 | 29418 | *rpoC2* | BE,FR,AP,IL,PE,PN |
| IL | dispersed | 39 | 45640 | 92945 | *ycf3*intron; *rps12_3-trnV*(GAC) | BE,FR,AP,IL,PE,PN |
| IL | dispersed | 31 | 82476 | 82521 | *rps3* | BE,FR,AP,IL,PE,PN |
| IL | panlindromic | 20 | 1 | 21 | *rps19-psbA* | BE,FR,AP,IL,PE,PN |
| IL | panlindromic | 20 | 17988 | 18654 | *trnD*(GUC)*-psbM; psbM-petN* | BE,FR,AP,IL,PE,PN |
| IL | panlindromic | 20 | 18167 | 18682 | *trnD*(GUC)*-psbM; psbM-petN* | AP,IL,PE,PN |
| IL | panlindromic | 22 | 18738 | 18764 | *psbM-petN* | AP,IL,PE,PN |
| IL | panlindromic | 21 | 44142 | 46320 | *psaA-ycf3; ycf3 intron* |  |
| IL | panlindromic | 22 | 73240 | 73265 | *psbT-psbN* | BE,FR,AP,IL,PE,PN |
| IL | panlindromic | 22 | 93664 | 94207 | *rps12_3-trnV*(GAC) | BE,FR,AP,IL,PE,PN |
| IL | panlindromic | 25 | 104268 | 104295 | *trnN*(GUU)*-rps15* | BE,FR,AP,IL,PE,PN |
| IL | panlindromic | 22 | 107544 | 108903 | *ndhF-rpl32; rpl32-trnL* | FR,AP,IL,PE,PN |
| IL | panlindromic | 20 | 107587 | 107923 | *ndhF-rpl32* | FR,AP,IL,PE,PN |
| IL | panlindromic | 21 | 107749 | 107884 | *ndhF-rpl32* | BE,FR,AP,IL,PE,PN |
| IL | panlindromic | 24 | 108661 | 108691 | *rpl32-trnL*(UAG) | BE,FR,AP,IL,PE,PN |
| PE | tandem | 16 | 16146 | 16162 | *trnG*(UCC)*-trnT*(GGU) | FR,AP,IL,PE,PN |
| PE | tandem | 16 | 20036 | 20052 | *petN-trnC*(GCA) | AP,IL,PE,PN |
| PE | tandem | 18 | 20933 | 20951 | *trnC*(GCA)*-rpoB* | BE,FR,AP,IL,PE,PN |
| PE | tandem | 21 | 27129 | 27150 | *rpoC1-rpoC2* | BE,AP,IL,PE,PN |
| PE | tandem | 15 | 28022 | 28037 | *rpoC2* | BE,FR,AP,IL,PE,PN |
| PE | tandem | 42 | 29224 | 29266 | *rpoC2* | AP,IL,PE,PN |
| PE | tandem | 18 | 61058 | 61076 | *ycf4-cemA* | BE,FR,AP,IL,PE,PN |
| PE | tandem | 17 | 68083 | 68100 | *rpl33-rps18* | FR,AP,IL,PE,PN |
| PE | tandem | 42 | 68228 | 68270 | *rps18* | FR,AP,IL,PE,PN |
| PE | tandem | 17 | 85907 | 85924 | *trnI*(CAU)*-trnL*(CAA) | BE,FR,AP,IL,PE,PN |
| PE | tandem | 16 | 105009 | 105025 | *rps15-ndhF* | PE,PN |
| PE | tandem | 31 | 108133 | 108164 | *ndhF-rpl32* | PE,PN |
| PE | tandem | 15 | 112465 | 112480 | *psaC-ndhE* | PE,PN |
| PE | tRNA similarity | 20 | 12974 | 13615 | *trnG*(UCC); *trnG*(UCC) | BE,FR,AP,IL,PE,PN |
| PE | tRNA similarity | 57 | 13453 | 38608 | *trnfM*(CAU); *trnfM*(CAU) | BE,FR,AP,IL,PE,PN |
| PE | gene similarity | 38 | 40666 | 42890 | *psaB; psaA* | BE,FR,AP,IL,PE,PN |
| PE | gene similarity | 32 | 58669 | 137313 | *rbcL-psaI; rpl23* | FR,AP,IL,PE,PN |
| PE | dispersed | 39 | 13404 | 38730 | *trnG*(UCC)*-trnfM*(CAU)*; trnfM*(CAU)-*rps14* | IL,PE,PN |
| PE | dispersed | 100 | 14505 | 84959 | *trnG*(UCC)-*trnT*(GGU)*;rpl2* | FR,AP,IL,PE,PN |
| PE | dispersed | 35 | 15163 | 92235 | *trnG*(UCC)*-trnT*(GGU)*; rps12_3 intron* | FR,AP,IL，PE,PN |
| PE | dispersed | 54 | 16333 | 16400 | *trnT*(GGU); *trnT*(GGU)*-trnE*(UUC) | FR,AP,IL,PE,PN |
| PE | dispersed | 111 | 29095 | 29296 | *rpoC2* | AP,IL,PE,PN |
| PE | dispersed | 39 | 29383 | 29449 | *rpoC2* | BE,FR,AP,IL,PE,PN |
| PE | dispersed | 39 | 45576 | 92885 | *ycf3*intron; *rps12_3-trnV*(GAC) | BE,FR,AP,IL,PE,PN |
| PE | dispersed | 31 | 82428 | 82473 | *rps3* | BE,FR,AP,IL,PE,PN |
| PE | panlindromic | 20 | 1 | 21 | *rps19-psbA* | BE,FR,AP,IL,PE,PN |
| PE | panlindromic | 20 | 18005 | 18671 | *trnD*(GUC)*-psbM; psbM-petN* | BE,FR,AP,IL,PE,PN |
| PE | panlindromic | 20 | 18184 | 18699 | *trnD*(GUC)*-psbM; psbM-petN* | AP,IL,PE,PN |
| PE | panlindromic | 22 | 18755 | 18781 | *psbM-petN* | AP,IL,PE,PN |
| PE | panlindromic | 20 | 31949 | 34521 | *rpoC2-rps2; atpI-atpH* | AP,PE,PN |
| PE | panlindromic | 20 | 73193 | 73218 | *psbT-psbN* | BE,FR,AP,IL,PE,PN |
| PE | panlindromic | 22 | 93604 | 94147 | *rps12_3-trnV*(GAC) | BE,FR,AP,IL,PE,PN |
| PE | panlindromic | 25 | 104214 | 104241 | *trnN*(GUU)-*rps15* | BE,FR,AP,IL,PE,PN |
| PE | panlindromic | 22 | 107506 | 108896 | *ndhF-rpl32; rpl32-trnL* | FR,AP,IL,PE,PN |
| PE | panlindromic | 20 | 107549 | 107885 | *ndhF-rpl32* | FR,AP,IL,PE,PN |
| PE | panlindromic | 21 | 107711 | 107846 | *ndhF-rpl32* | BE,FR,AP,IL,PE,PN |
| PE | panlindromic | 24 | 108654 | 108684 | *rpl32-trnL*(UAG) | BE,FR,AP,IL,PE,PN |
| PN | tandem | 16 | 16139 | 16155 | *trnG*(UCC)*-trnT*(GGU) | FR,AP,IL,PE,PN |
| PN | tandem | 16 | 20017 | 20033 | *petN-trnC*(GCA) | AP,IL,PE,PN |
| PN | tandem | 18 | 20914 | 20932 | *trnC*(GCA)*-rpoB* | BE,FR,AP,IL,PE,PN |
| PN | tandem | 21 | 27110 | 27131 | *rpoC1-rpoC2* | BE,AP,IL,PE,PN |
| PN | tandem | 15 | 28003 | 28018 | *rpoC2* | BE,FR,AP,IL,PE,PN |
| PN | tandem | 42 | 29205 | 29247 | *rpoC2* | AP,IL,PE,PN |
| PN | tandem | 37 | 59075 | 59112 | *rbcL-psaI* |  |
| PN | tandem | 18 | 61067 | 61085 | *ycf4-cemA* | BE,FR,AP,IL,PE,PN |
| PN | tandem | 17 | 68092 | 68109 | *rpl33-rps18* | FR,AP,IL,PE,PN |
| PN | tandem | 42 | 68236 | 68278 | *rps18* | FR,AP,IL,PE,PN |
| PN | tandem | 17 | 85928 | 85945 | *trnI*(CAU)*-trnL*(CAA) | BE,FR,AP,IL,PE,PN |
| PN | tandem | 65 | 100376 | 100441 | *rrn23* |  |
| PN | tandem | 16 | 105095 | 105111 | *rps15-ndhF* | PE,PN |
| PN | tandem | 31 | 108219 | 108250 | *ndhF-rpl32* | PE,PN |
| PN | tandem | 15 | 112550 | 112565 | *psaC-ndhE* | PE,PN |
| PN | tRNA similarity | 57 | 13446 | 38589 | *trnfM*(CAU); *trnfM*(CAU) | BE,FR,AP,IL,PE,PN |
| PN | tRNA similarity | 20 | 12967 | 13608 | *trnG*(UCC); *trnG*(UCC) | BE,FR,AP,IL,PE,PN |
| PN | gene similarity | 38 | 40647 | 42871 | *psaB; psaA* | BE,FR,AP,IL,PE,PN |
| PN | gene similarity | 32 | 58642 | 137473 | *rbcL-psaI; rpl23* | FR,AP,IL,PE,PN |
| PN | dispersed | 39 | 13397 | 38711 | *trnG*(UCC)*-trnfM*(CAU); *trnfM*(CAU)*-rps14* | IL,PE,PN |
| PN | dispersed | 100 | 14498 | 84980 | *trnG*(UCC)*-trnT*(GGU)*;rpl2* | FR,AP,IL,PE,PN |
| PN | dispersed | 35 | 15156 | 92256 | *trnG*(UCC)*-trnT*(GGU)*; rps12_3* intron | FR,AP,IL,PE,PN |
| PN | dispersed | 54 | 16326 | 16393 | *trnT*(GGU); *trnT*(GGU)*-trnE*(UUC) | FR,AP,IL,PE,PN |
| PN | dispersed | 111 | 29076 | 29277 | *rpoC2* | AP,IL,PE,PN |
| PN | dispersed | 39 | 29364 | 29430 | *rpoC2* | BE,FR,AP,IL,PE,PN |
| PN | dispersed | 39 | 45548 | 92906 | *ycf3*intron; *rps12_3-trnV*(GAC) | BE,FR,AP,IL,PE,PN |
| PN | dispersed | 31 | 82437 | 82482 | *rps3* | BE,FR,AP,IL,PE,PN |
| PN | panlindromic | 20 | 1 | 21 | *rps19-psbA* | BE,FR,AP,IL,PE,PN |
| PN | panlindromic | 20 | 18000 | 18666 | *trnD*(GUC)*-psbM; psbM-petN* | BE,FR,AP,IL,PE,PN |
| PN | panlindromic | 20 | 18179 | 18694 | *trnD*(GUC)*-psbM; psbM-petN* | AP,IL,PE,PN |
| PN | panlindromic | 22 | 18750 | 18776 | *psbM-petN* | AP,IL,PE,PN |
| PN | panlindromic | 20 | 31930 | 34502 | *rpoC2-rps2; atpI-atpH* | AP,PE,PN |
| PN | panlindromic | 20 | 73201 | 73226 | *psbT-psbN* | BE,FR,AP,IL,PE,PN |
| PN | panlindromic | 22 | 93625 | 94168 | *rps12_3-trnV*(GAC) | BE,FR,AP,IL,PE,PN |
| PN | panlindromic | 25 | 104300 | 104327 | *trnN*(GUU)*-rps15* | BE,FR,AP,IL,PE,PN |
| PN | panlindromic | 22 | 107592 | 108982 | *ndhF-rpl32; rpl32-trnL* | FR,AP,IL,PE,PN |
| PN | panlindromic | 20 | 107635 | 107971 | *ndhF-rpl32* | FR,AP,IL,PE,PN |
| PN | panlindromic | 21 | 107797 | 107932 | *ndhF-rpl32* | BE,FR,AP,IL,PE,PN |
| PN | panlindromic | 24 | 108740 | 108770 | *rpl32-trnL*(UAG) | BE,FR,AP,IL,PE,PN |

**^A^** BE, *B. emeiensis*; FR, *F. rimosivaginus*; AP, *A. purpurea*; IL, *I. longiauritus*; PE, *P. edulis*; PN, *P. nigra* var. *henonis*.
