# Supplementary material for: High-Throughput Sequencing of Six Bamboo Chloroplast Genomes: Phylogenetic Implications for Temperate Woody Bamboos (Poaceae: Bambusoideae)
Source: PLoS One. 2011 May 31;6(5):e20596. doi: 10.1371/journal.pone.0020596 (PMC3105084; doi:10.1371/journal.pone.0020596)
Supplement: Table S4 — The locations and sequences of 12 small inversions in eight woody bamboo chloroplast genomes. (DOC) [file pone.0020596.s006.doc]

**Table S4.** The locations and sequences of 12 small inversions in eight woody bamboo chloroplast genomes.

| **No.** | **Locus** | **Taxon^A^** | **Genome Location** | **Sequence Alignment** | **Free Energy (∆G)** |
| --- | --- | --- | --- | --- | --- |
| 1 | *rps19-psbA* | DL | 28-67 | AAATACCCAATATCTTG**TTCCAG**CAAGATATTGGGTATTT | -15.94 |
|  |  | BO | 5-44 | AAATACCCAATATCTTG**CTGGAA**CAAGATATTGGGTATTT | -15.35 |
|  |  | BE | 5-44 | AAATACCCAATATCTTG**CTGGAA**CAAGATATTGGGTATTT | -15.35 |
|  |  | FR | 1-40 | AAATACCCAATATCTTG**CTGGAA**CAAGATATTGGGTATTT | -15.35 |
|  |  | AP | 1-40 | AAATACCCAATATCTTG**CTGGAA**CAAGATATTGGGTATTT | -15.35 |
|  |  | IL | 1-40 | AAATACCCAATATCTTG**CTGGAA**CAAGATATTGGGTATTT | -15.35 |
|  |  | PE | 1-40 | AAATACCCAATATCTTG**CTGGAA**CAAGATATTGGGTATTT | -15.35 |
|  |  | PN | 1-40 | AAATACCCAATATCTTG**CTGGAA**CAAGATATTGGGTATTT | -15.35 |
| 2 | *psbK-psbI* | DL | 7,555-7,590 | AAATCAATTCTTG**AAAAAAATTG**CAAGAATTGATTT | -10.00 |
|  |  | BO | 7,527-7,562 | AAATCAATTCTTG**AAAAAAATTG**CAAGAATTGATTT | -10.00 |
|  |  | BE | 7,530-7,565 | AAATCAATTCTTG**AAAAAAATTG**CAAGAATTGATTT | -10.00 |
|  |  | FR | 8,013-8,048 | AAATCAATTCTTG**CAATTTTTTT**CCAGAATTTATTT | -2.27 |
|  |  | AP | 8,016-8,051 | AAATCAATTCTTG**CAATTTTTTT**CCAGAATTGATTT | -6.74 |
|  |  | IL | 8,014-8,049 | AAATCAATTCTTG**CAATTTTTTT**CAAGAATTGATTT | -6.74 |
|  |  | PE | 8,016-8,051 | AAATCAATTCTTG**CAATTTTTTT**CAAGAATTGATTT | -6.74 |
|  |  | PN | 8,008-8,043 | AAATCAATTCTTG**CAATTTTTTT**CAAGAATTGATTT | -6.74 |
|  |  | FR | 8,013-8,048 | AAATCAATTCTTG**CAATTTTTTT**CCAGAATTTATTT | -2.27 |
| 3 | *rbcL-psaI* | DL | 58,305-58,338 | TCGGCTCAATC**TTTTTTTTTTAA**GATTGAGCCGA | -13.24 |
|  |  | BO | 58,187-58,219 | TCGGCTCAATC**-TTTTTTTTTAA**GATTGAGCCGA | -13.24 |
|  |  | BE | 58,292-58,324 | TCGGCTCAATC**-TTTTTTTTTAA**GATTGAGCCGA | -13.24 |
|  |  | FR | 58,251-58,282 | TCGGCTCAATC**--TTAAAAAAAA**GATTGAGCCGA | -13.84 |
|  |  | AP | 58,565-58,596 | TCGGCTCAATC**--TTTTTTTTAA**GATTGAGCCGA | -13.54 |
|  |  | IL | 58,572-58,603 | TCGGCTCAATC**--TTAAAAAAAA**GATTGAGCCGA | -13.84 |
|  |  | PN | 58,487-58,518 | TCGGCTCAATC**--TTAAAAAAAA**GATTGAGCCGA | -13.84 |
| 4 | *rbcL-psaI* | DL | 58,421-58,463 | AATACAATAAATAAATAC**TATATTT**GTATTTATTTATTGTATT | -12.72 |
|  |  | BO | 58,302-58,344 | AATACAATAAATAAATAC**AAATATA**GTATTTATTTATTGTATT | -12.82 |
|  |  | BE | 58,407-58,449 | AATACAATAAATAAATAC**TATATTT**GTATTTATTTATTGTATT | -12.72 |
|  |  | FR | 58,365-58,394 | AATACAATAAATAAATAC**AAATATA**GTATT------------- | -1.06 |
|  |  | AP | 58,679-58,708 | AATACAATAAATAAATAC**AAATATA**GTATT------------- | -1.06 |
|  |  | IL | 58,686-58,715 | AATACAATAAATAAATAC**AAATATA**GTATT------------- | -1.06 |
|  |  | PE | 58,628-58,657 | AATACAATAAATAAATAC**AAATATA**GTATT------------- | -1.06 |
|  |  | PN | 58,601-58,630 | AATACAATAAATAAATAC**AAATATA**GTATT------------- | -1.06 |
| 5 | *petA-psbJ* | DL | 63,016-63,049 | CACAAGAAAAAGGC**TTTTTT**GCCTTTTTCTTGTG | -14.18 |
|  |  | BO | 62,909-62,942 | CACAAGAAAAAGGC**TTTTTT**GCCTTTTTCTTGTG | -14.18 |
|  |  | BE | 63,015-63,048 | CACAAGAAAAAGGC**TTTTTT**GCCTTTTTCTTGTG | -14.18 |
|  |  | FR | 62,992-63,025 | CACAAGAAAAAGGC**AAAAAA**GCCTTTTTCTTGTG | -14.28 |
|  |  | AP | 63,297-63,330 | CACAAGAAAAAGGC**TTTTTT**GCCTTTTTCTTGTG | -14.18 |
|  |  | IL | 63,291-63,324 | CACAAGAAAAAGGC**TTTTTT**GCCTTTTTCTTGTG | -14.18 |
|  |  | PE | 63,244-63,277 | CACAAGAAAAAGGC**TTTTTT**GCCTTTTTCTTGTG | -14.18 |
|  |  | PN | 63,253-63,286 | CACAAGAAAAAGGC**TTTTTT**GCCTTTTTCTTGTG | -14.18 |
| 6 | *rpl20-rps12_5* | DL | 69,646-69,668 | TAGGATAGAG**CTT**CTCTATCCTA | -7.38 |
|  |  | BO | 69,544-69,566 | TAGGATAGAG**CTT**CTCTATCCTA | -7.38 |
|  |  | BE | 69,644-69,666 | TAGGATAGAG**CTT**CTCTATCCTA | -7.38 |
|  |  | FR | 69,664-69,686 | TAGGATAGAG**AAG**CTCTATCCTA | -7.38 |
|  |  | AP | 69,929-69,951 | TAGGATAGAG**AAG**CTCTATCCTA | -7.38 |
|  |  | IL | 69,931-69,953 | TAGGATAGAG**AAG**CTCTATCCTA | -7.38 |
|  |  | PE | 69,886-69,908 | TAGGATAGAG**AAG**CTCTATCCTA | -7.38 |
|  |  | PN | 69,894-69,916 | TAGGATAGAG**AAG**CTCTATCCTA | -7.38 |
| 7 | *psbT-psbN* | DL | 72,962-73,006 | ATTGAAGTAAGAAGTCTCCC**CATCT**GGGAGACTTCTTACTTCAAT | -21.14 |
|  |  | BO | 72,860-72,904 | ATTGAAGTAAGAAGTCTCCC**CATCT**GGGAGACTTCTTACTTCAAT | -21.14 |
|  |  | BE | 72,962-73,006 | ATTGAAGTAAGAAGTCTCCC**CATCT**GGGAGACTTCTTACTTCAAT | -21.14 |
|  |  | FR | 72,978-73,022 | ATTGAAGTAAGAAGTCTCCC**AGATG**GGGAGACTTCTTACTTCAAT | -21.34 |
|  |  | AP | 73,235-73,279 | ATTGAAGTAAGAAGTCTCCC**AGATG**GGGAGACTTCTTACTTCAAT | -21.34 |
|  |  | IL | 73,240-73,284 | ATTGAAGTAAGAAGTCTCCC**AGATG**GGGAGACTTCTTACTTCAAT | -21.34 |
|  |  | PE | 73,193-73,237 | ATTGAAGTAAGAAGTCTCCC**AGATG**GGGAGACTTCTTACTTCAAT | -21.34 |
|  |  | PN | 73,201-73,245 | ATTGAAGTAAGAAGTCTCCC**AGATG**GGGAGACTTCTTACTTCAAT | -21.34 |
| 8 | *petD-rpoA* | DL | 76,525-76,574 | TAGGTATCTAGG**AAATAGTTACTTCCAAGTGAATCTTC**CCTAGATACCTA | -9.36 |
|  |  | BO | 76,424-76,473 | TAGGTATCTAGG**AAATAGTTACTTCCAAGTGAATCTTC**CCTAGATACCTA | -9.36 |
|  |  | BE | 76,526-76,575 | TAGGTATCTAGG**AAATAGTTACTTCCAAGTGAATCTTC**CCTAGATACCTA | -9.36 |
|  |  | FR | 76,544-76,593 | TAGGTATCTAGG**AAATAGTTACTTCCAAGTGAATCTTC**CCTAGATACCTA | -9.36 |
|  |  | AP | 76,792-76,841 | TAGGTATCTAGG**GAAGATTCACTTGGAAGTAACTATTT**CCTAGATACCTA | -10.14 |
|  |  | IL | 76,803-76,852 | TAGGTATCTAGG**AAATAGTTACTTCCAAGTGAATCTTC**CCTAGATACCTA | -9.36 |
|  |  | PE | 76,756-76,805 | TAGGTATCTAGG**AAATAGTTACTTCCAAGTGAATCTTC**CCTAGATACCTA | -9.36 |
|  |  | PN | 76,763-76,812 | TAGGTATCTAGG**AAATAGTTACTTCCAAGTGAATCTTC**CCTAGATACCTA | -9.36 |
| 9 | *rpl16* intron | DL | 80,608-80,633 | TCCATAATATT**TTGG**AATATTATGGA | -7.69 |
|  |  | BO | 80,510-80,535 | TCCATAATATT**CCAA**AATATTATGGA | -7.59 |
|  |  | BE | 80,612-80,637 | TCCATAATATT**TTGG**AATATTATGGA | -7.69 |
|  |  | FR | 80,652-80,677 | TCCATAATATT**TTGG**AATATTATGGA | -7.69 |
|  |  | AP | 80,891-80,916 | TCCATAATATT**TTGG**AATATTATGGA | -7.69 |
|  |  | IL | 80,874-80,899 | TCCATAATATT**TTGG**AATATTATGGA | -7.69 |
|  |  | PE | 80,826-80,851 | TCCATAATATT**TTGG**AATATTATGGA | -7.69 |
|  |  | PN | 80,835-80,860 | TCCATAATATT**TTGG**AATATTATGGA | -7.69 |
| 10 | *trnN*(GUU)*-rps15* | DL | 103,958-104,009 | ATTCTTTTATTTTAGATAGAAGAAA**CA**TTTCTTCTATCTAAAATAAAAGAAT | -21.05 |
|  |  | BO | 103,872-103,923 | ATTCTTTTATTTTAGATAGAAGAAA**CA**TTTCTTCTATCTAAAATAAAAGAAT | -21.05 |
|  |  | BE | 103,983-104,034 | ATTCTTTTATTTTAGATAGAAGAAA**CA**TTTCTTCTATCTAAAATAAAAGAAT | -21.05 |
|  |  | FR | 104,123-104,174 | ATTCTTTTATTTTAGATAGAAGAAA**TG**TTTCTTCTATCTAAAATAAAAGAAT | -21.05 |
|  |  | AP | 104,271-104,322 | ATTCTTTTATTTTAGATAGAAGAAA**TG**TTTCTTCTATCTAAAATAAAAGAAT | -21.05 |
|  |  | IL | 104,268-104,319 | ATTCTTTTATTTTAGATAGAAGAAA**TG**TTTCTTCTATCTAAAATAAAAGAAT | -21.05 |
|  |  | PE | 104,214-104,265 | ATTCTTTTATTTTAGATAGAAGAAA**TG**TTTCTTCTATCTAAAATAAAAGAAT | -21.05 |
|  |  | PN | 104,300-104,351 | ATTCTTTTATTTTAGATAGAAGAAA**TG**TTTCTTCTATCTAAAATAAAAGAAT | -21.05 |
| 11 | ***rpl32-trnL*(UAG)** | DL | 108,365-108,418 | ACTTTTCATAATAGAATCCTCATA**TTTTAT**TATGAGGATTCTATTATGAAAAGT | -21.83 |
|  |  | BO | 108,292-108,345 | ACTTTTCATAATAGAATCCTCATA**ATAAAA**TATGAGGATTCTATTATGAAAAGT | -22.13 |
|  |  | BE | 108,426-108,479 | ACTTTTCATAATAGAATCCTCATA**TTTTAT**TATGAGGATTCTATTATGAAAAGT | -21.83 |
|  |  | FR | 108,413-108,466 | ACTTTTCATAATAGAATCCTCATA**TTTTAT**TATGAGGATTCTATTATGAAAAGT | -21.83 |
|  |  | AP | 108,698-108,751 | ACTTTTCATAATAGAATCCTCATA**ATAAAA**TATGAGGATTCTATTATGAAAAGT | -22.13 |
|  |  | IL | 108,661-108,714 | ACTTTTCATAATAGAATCCTCATA**ATAAAA**TATGAGGATTCTATTATGAAAAGT | -22.13 |
|  |  | PE | 108,654-108,707 | ACTTTTCATAATAGAATCCTCATA**TTTTAT**TATGAGGATTCTATTATGAAAAGT | -21.83 |
|  |  | PN | 108,740-108,793 | ACTTTTCATAATAGAATCCTCATA**ATAAAA**TATGAGGATTCTATTATGAAAAGT | -22.13 |
| 12 | *ndhD-psaC* | DL | 111,777-111,809 | AAGCCCGTGCTCG**CTTATTT**CGAGCACAGGCTT | -11.05 |
|  |  | BO | 111,706-111,738 | AAGCCCGTGCTCG**CTTATTT**CGAGCACAGGCTT | -11.05 |
|  |  | BE | 111,838-111,870 | AAGCCCGTGCTCG**CTTATTT**CGAGCACAGGCTT | -11.05 |
|  |  | FR | 111,778-111,810 | AAGCCCGTGCTCG**AAATAAG**CGAGCACAGGCTT | -11.15 |
|  |  | AP | 112,063-112,095 | AAGCCCGTGCTCG**AAATAAG**CGAGCACAGGCTT | -11.15 |
|  |  | IL | 112,024-112,056 | AAGCCCGTGCTCG**AAATAAG**CGAGCACAGGCTT | -11.15 |
|  |  | PE | 112,018-112,050 | AAGCCCGTGCTCG**AAATAAG**CGAGCACAGGCTT | -11.15 |
|  |  | PN | 112,103-112,135 | AAGCCCGTGCTCG**AAATAAG**CGAGCACAGGCTT | -11.15 |

**^A^** DL, *D. latiflorus*; BO, *B. oldhamii*; BE, *B. emeiensis*; FR, *F. rimosivaginus*; AP, *A. purpurea*; IL, *I. longiauritus*; PE, *P. edulis*; PN, *P. nigra* var. *henonis*.
