# Supplementary material for: High-Throughput Sequencing of Six Bamboo Chloroplast Genomes: Phylogenetic Implications for Temperate Woody Bamboos (Poaceae: Bambusoideae)
Source: PLoS One. 2011 May 31;6(5):e20596. doi: 10.1371/journal.pone.0020596 (PMC3105084; doi:10.1371/journal.pone.0020596)
Supplement: Table S5 — Indels in exons of 21 genes in the grass chloroplast genomes. (DOC) [file pone.0020596.s007.doc]

**Table S5. Indels in exons of 21 genes in the grass chloroplast genome.**

| **Gene** | **Homoplasious** | **Synapomorphic** | **Total** |
| --- | --- | --- | --- |
| *atpA* | 0 | 1 | 1 |
| *ccsA* | 2 | 2 | 4 |
| *infA* | 1 | 0 | 1 |
| *matK* | 1 | 1 | 2 |
| *ndhD* | 0 | 1 | 1 |
| *ndhF* | 1 | 2 | 3 |
| *ndhK* | 1 | 1 | 2 |
| *psbT* | 1 | 1 | 2 |
| *rbcL* | 0 | 2 | 2 |
| *rpl16* | 0 | 1 | 1 |
| *rpl22* | 0 | 1 | 1 |
| *rpl32* | 1 | 0 | 1 |
| *rpoA* | 0 | 2 | 2 |
| *rpoC1* | 1 | 1 | 2 |
| *rpoB* | 0 | 1 | 1 |
| *rpoC2* | 5 | 5 | 10 |
| *rps3* | 1 | 1 | 2 |
| *rps12* | 1 | 0 | 1 |
| *rps15* | 1 | 0 | 1 |
| *rps16* | 0 | 1 | 1 |
| *rps18* | 3 | 1 | 4 |
