# Supplementary material for: High-Throughput Sequencing of Six Bamboo Chloroplast Genomes: Phylogenetic Implications for Temperate Woody Bamboos (Poaceae: Bambusoideae)
Source: PLoS One. 2011 May 31;6(5):e20596. doi: 10.1371/journal.pone.0020596 (PMC3105084; doi:10.1371/journal.pone.0020596)
Supplement: Table S6 — Summary statistics for noncoding regions in the chloroplast genomes of Bambusoideae. (DOC) [file pone.0020596.s008.doc]

**Table S6.** Summary statistics for noncoding regions in the chloroplast genomes of Bambusoideae.

| Region^A^ | Aligned length (bp) | No. of variable sites (indels) | Percentage of variable sites | No. of PICs (indels) | Percentage of PICs |
| --- | --- | --- | --- | --- | --- |
| ***trnD*(GUC)*-psbM*** | 1094 | 71 (18) | 8.135 | 33 (6) | 3.565 |
| ***ycf4-cemA*** | 459 | 28 (7) | 7.625 | 14 (3) | 3.704 |
| ***trnG*(UCC)*-trnT*(GGU)** | 2125 | 129 (22) | 7.106 | 96 (10) | 4.988 |
| ***ndhF-rpl32*** | 1001 | 57 (12) | 6.893 | 36 (5) | 4.096 |
| ***rpl32-trnL*(UAG)*** | 736 | 44 (3) | 6.386 | 30 (1) | 4.212 |
| ***trnK*(UUU*)-rps16**** | 557 | 29 (3) | 5.745 | 21 (1) | 3.950 |
| ***psbK-psbI*** | 416 | 19 (4) | 5.529 | 16 (2) | 4.327 |
| ***ycf3-trnS*(GGA)** | 598 | 18 (14) | 5.351 | 10 (1) | 1.839 |
| ***trnT*(UGU)*-trnL*(UAA)*** | 838 | 35 (6) | 4.893 | 27 (1) | 3.341 |
| ***psbZ-trnfM*(CAU)** | 830 | 29 (10) | 4.699 | 20 (5) | 3.012 |
| ***rbcL-psaI*** | 1346 | 51 (10) | 4.532 | 36 (5) | 3.046 |
| ***psaC-ndhE*** | 536 | 16 (8) | 4.478 | 13 (5) | 3.358 |
| ***trnT*(GGU)*-trnE*(UUC)*** | 559 | 18 (7) | 4.472 | 12 (4) | 2.862 |
| ***trnY*(GUA)*-trnD*(GUC)*** | 358 | 12 (3) | 4.190 | 8 (3) | 3.073 |
| ***rps15-ndhF*** | 504 | 18 (3) | 4.167 | 8 (1) | 1.786 |
| ***trnL*(UAA)*-trnF*(GAA)** | 362 | 10 (5) | 4.144 | 10 (2) | 3.315 |
| ***trnF*(GAA)*-ndhJ*** | 585 | 22 (2) | 4.103 | 13 (1) | 2.393 |
| ***rpl16* intron** | 1131 | 38 (8) | 4.067 | 17 (5) | 1.945 |
| ***psaI-ycf4*** | 370 | 13 (2) | 4.054 | 12 (1) | 3.514 |
| ***psaA-ycf3**** | 646 | 18 (8) | 4.025 | 14 (2) | 2.477 |
| *psaJ-rpl33* | 473 | 16 (3) | 4.017 | 9 | 1.903 |
| *petD* intron | 754 | 25 (4) | 3.846 | 14 (2) | 2.122 |
| *psbM-petN* | 782 | 25 (4) | 3.708 | 17 (2) | 2.430 |
| *trnL*(UAA) intron | 546 | 16 (4) | 3.663 | 12 (3) | 2.747 |
| *petN-trnC*(GCA) | 935 | 27 (7) | 3.636 | 23 (2) | 2.674 |
| *petA-psbJ* | 1019 | 29 (8) | 3.631 | 20 (5) | 2.453 |
| *psbE-petL* | 1235 | 38 (6) | 3.563 | 29 (5) | 2.753 |
| *trnS*(GCU)*-psbD* | 1022 | 30 (6) | 3.523 | 21 (1) | 2.153 |
| *trnP*(UGG)*-psaJ* | 410 | 10 (4) | 3.415 | 7 (3) | 2.439 |
| *trnC*(GCA)*-rpoB** | 1217 | 31 (10) | 3.369 | 23 (5) | 2.301 |
| *ndhC-trnV*(UAC)* | 972 | 24 (7) | 3.189 | 16 (4) | 2.058 |
| *NdhA* intron | 1026 | 28 (4) | 3.119 | 23 (3) | 2.534 |
| *rps16-trnQ*(UUG)* | 1551 | 39 (9) | 3.095 | 27 (5) | 2.063 |
| *petB* intron | 832 | 18 (7) | 3.005 | 14 (3) | 2.043 |
| *trnG*(UCC) intron* | 673 | 18 (1) | 2.823 | 12 | 1.783 |
| *atpF* intron | 846 | 15 (8) | 2.719 | 5 (3) | 0.946 |
| *trnH-psbA** | 566 | 10 (5) | 2.650 | 5 (3) | 1.413 |
| *atpH-atpF* | 457 | 10 (2) | 2.626 | 8 (1) | 1.969 |
| *trnS*(UGA)*-psbZ* | 350 | 6 (3) | 2.571 | 2 (3) | 1.429 |
| *trnQ*(UUG)*-psbK* | 351 | 8 (1) | 2.564 | 5 (1) | 1.709 |
| *atpB-rbcL* | 788 | 15 (4) | 2.411 | 9 (4) | 1.650 |
| *atpI-atpH** | 871 | 14 (6) | 2.296 | 11 (4) | 1.722 |
| *rps16* intron* | 846 | 18 (1) | 2.246 | 13 (1) | 1.655 |
| *clpP-psbB* | 520 | 8 (3) | 2.115 | 6 | 1.154 |
| *ycf3* intron1 | 734 | 10 (5) | 2.044 | 6 (2) | 1.090 |
| *rpl20-rps12_5* | 697 | 12 (2) | 2.009 | 7 (2) | 1.291 |
| *trnV(UAC)* intron | 604 | 5 (4) | 1.490 | 2 | 0.331 |
| *ycf3* intron2 | 751 | 8 (2) | 1.332 | 4 (1) | 0.666 |

^A^ Fragments rank from most variable to least variable. The top 20 fragments are indicated in boldface.

* Fragments have been used in previous phylogenetic studies in Bambusoideae.

PICs: parsimony-informative characters.
